# Experience and rationality under risk: re-examining the impact of sampling experience

**DOI:** 10.1007/s10683-019-09641-y

**Published:** 2020-01-16

**Authors:** Ilke Aydogan, Yu Gao

**Affiliations:** 1 IESEG School of Management, Lille, France; 2 Department of Economics, Bocconi University, Milan, Italy; 3 Department of Applied Economics, Guanghua School of Management (GSM), Peking University, Beijing, 100871 China

**Keywords:** Decisions from experience, Decisions under risk, Probability weighting, Rare outcomes, D81, D83, C91

## Abstract

A recent strand of the literature on decision-making under uncertainty has pointed to an intriguing behavioral gap between decisions made from description and decisions made from experience. This study reinvestigates this description-experience gap to understand the impact that sampling experience has on decisions under risk. Our study adopts a complete sampling paradigm to address the lack of control over experienced probabilities by requiring complete sampling without replacement. We also address the roles of utilities and ambiguity, which are central in most current decision models in economics. Thus, our experiment identifies the deviations from expected utility due to over- (or under-) weighting of probabilities. Our results confirm the existence of the behavioral gap, but they provide no evidence for the underweighting of small probabilities within the complete sampling treatment. We find that sampling experience attenuates rather than reverses the inverse S-shaped probability weighting under risk.

**Electronic supplementary material:**

The online version of this article (10.1007/s10683-019-09641-y) contains supplementary material, which is available to authorized users.

The traditional paradigm of decisions from description (DFD), which uses explicit descriptions of probability distributions over outcomes, has served for decades as a useful tool for studying decision-making under risk in the laboratory. This paradigm has led to important empirical findings on systematic deviations from expected utility theory (EU) (von Neumann and Morgenstern 1944; Allais 1953; Tversky and Kahneman 1981) and has given rise to significant theoretical developments, including prospect theory (PT) (Kahneman and Tversky 1979; Tversky and Kahneman 1992). Among these developments, non-linearity of decision weights in probabilities has been acknowledged as one of the most important deviations from EU. The famous inverse S-shaped probability weighting, which captures the tendency to overweight rare and extreme outcomes in prospects, is the most commonly documented pattern in numerous laboratory studies. It also provides a useful framework for understanding and predicting field behavior in financial, insurance, and betting markets that cannot be explained by EU (Fehr-Duda and Epper 2012).

The predominant view on inverse S-shaped probability weighting driven by the DFD paradigm has been challenged by a recent strand of literature, which has mainly arisen in the field of psychology. The studies by Barron and Erev (2003) and Hertwig et al. (2004) argued that DFD fails to represent many real-life decisions. In particular, DFD cannot explain decisions in which people do not have complete descriptions of the prospects before them, and they have to rely on their past experiences. Therefore, these studies have introduced an alternative experimental paradigm, which is called decisions from experience (DFE). In the DFE paradigm, subjects learn about outcomes and probabilities by drawing samples from underlying probability distributions, usually with replacement. Importantly, the findings in these studies suggest that some of the common choice patterns that violate EU (e.g., the common ratio effect) are reversed under DFE. In particular, people make decisions from experience *as if* they are underweighting rare and extreme outcomes. Notwithstanding the findings observed under DFD, the underweighting of rare and extreme outcomes in DFE has been claimed to be one of the factors that cause failures of risk management in the financial industry (Hertwig and Erev 2009; Taleb 2007).

The intriguing choice discrepancy between the DFD and DFE paradigms (or the so-called description-experience gap) has received considerable attention in studies of both psychology and economics (Palma et al. 2014; Hertwig 2012). The accumulated body of literature on DFE has confirmed that the description-experience gap is substantial (see the meta-analysis by Wulff et al. (2018)). However, as robust as this discrepancy in choice behavior stands, its implications for probability weighting have remained unclear. In particular, it remains undetermined whether sampling experience can result in other deviations from EU by reversing the common patterns observed under DFD or if it only attenuates the prevailing deviations. Indeed, the attenuating effects of experience have been commonly addressed in experimental tests of EU, as reported in the economics literature (see Sect. 2.6 in Bardsley et al. (2010)). A proper understanding of the precise impact of experience (reversing or reducing irrationalities) is essential for finding appropriate applications of the standard theory of rational choice, and for understanding and predicting economic behavior. The objective of this study is to re-consider the description-experience gap by focusing on the role of probability weighting, and to provide a valid test of the deviations from EU that occur in the presence of sampling experience.

Our study addresses several issues related to the measurement of probability weighting under DFE. First, we acknowledge that early studies in the DFE literature originally introduced the description-experience gap as a discrepancy in choice behavior. The initial conclusions on underweighting in DFE were drawn in an “as if” sense, as a way of referring to choice propensities toward either risk-aversion or risk-seeking, rather than assessing such propensities by measuring the components involved in PT. For example, an underweighting of 10% probability was typically inferred from a majority preference for a sure $1 prize over a lottery with a 10% chance of winning $10 (and a 90% chance of getting $0). This approach left the link between choice behavior and the actual weighting of probabilities unclear, as a proper measurement of utilities is required for valid inferences about probability weightings. More recent studies of DFE have included attempts to use parametric estimations of PT components (see Sect. 2.2).

The second issue concerns a kind of information asymmetry between DFD and DFE (Hadar and Fox 2009). DFD and DFE differ not only in their processes of information acquisition (i.e., through description or by experience) but also in terms of information available to the decision-maker. DFD represents a case of risk, where the outcome probabilities are known. DFE, on the other hand, represents a case of ambiguity in which the outcome probabilities, and even the set of possible outcomes may be unknown. Therefore, when making comparisons between DFD and DFE, the impact of experience potentially interacts with well-known attitudes toward unknown probabilities (Ellsberg 1961; Trautmann and Van De Kuilen 2015). Furthermore, when the set of possible outcomes is unknown, this ambiguity poses a problem for testing EU and non-EU theories, as having a well-defined set of potential outcomes is usually taken as primitive in these theories.

Our experiment addresses the issue of information asymmetry by adapting the original sampling paradigm proposed by Hertwig et al. (2004). In particular, we use a complete sampling paradigm (CSP), which requires that our subjects experience the precise objective probabilities by sampling fully without replacement. Thus, our description and sampling treatments represent two different cases of risk, in which information on objective probabilities is provided in different ways. Although this approach departs from the original paradigm of DFE, the regulation of sampling experiences in a CSP design is helpful for a clean measurement of probability weighting, as is explained in Sect. 2.1. This approach, therefore, enables us to draw new insights from DFE.

In addition, our experiment applies a robust two-stage methodology to measure probability weighting (Abdellaoui 2000; Bleichrodt and Pinto 2000; Etchart-Vincent 2004, 2009; Qiu and Steiger 2010). Specifically, we measure utilities in the first stage, and then observe the direct links between observed risky choices and the actual over- or underweighting of probabilities in the second stage. Hence, we identify the direction and the magnitude of the deviations from EU in a nonparametric way, without relying on any parametric assumptions about probability weighting. We also run parametric estimations by using Bayesian hierarchical modeling as a supplement to our nonparametric measures.

## Deviations from EU due to probability weighting

We restrict our attention to probability-contingent binary prospects in the gain domain. A binary prospect of winning *x* with probability *p* and *y* otherwise is denoted as $x_{p} y$. Under rank-dependent utility theory (RDU), for $x\, \succcurlyeq \, y\, \succcurlyeq\, 0,\, x_{p} y$ is evaluated by $w\left( p \right)U\left( x \right) + \left( {1 - w\left( p \right)} \right)U\left( y \right)$ where *U* is the utility function and *w* the probability weighting function. Throughout our tests, we assume a binary RDU. Most other non-EU theories, and particularly both versions of PT for gains (Kahneman and Tversky 1979; Tversky and Kahneman 1992), and disappointment aversion theory (Gul 1991), all agree with the binary RDU in the evaluation of binary prospects (Observation 7.11.1 in Wakker 2010, pp. 231). Hence, our analysis applies to all of these theories.

**Fig. 1:**
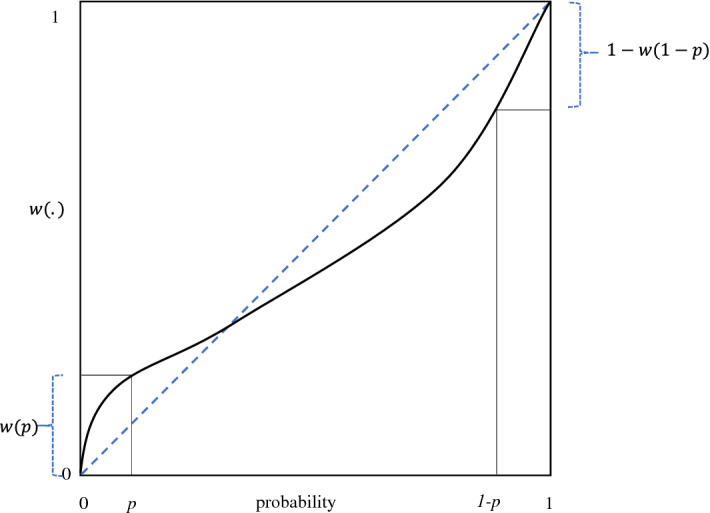
Inverse S-shaped probability weighting function

RDU deviates from EU when $w\left( . \right)$ is not the identity. Thus, a decision maker’s attitude toward risk depends not only on the utility curvature (as in EU), but also on the probability weighting. Figure 1 illustrates an inverse S-shaped probability weighting function, which is first concave and overweighting, and then convex and underweighting.^1^ The steepness of the probability weighting function at both endpoints implies that in general, the rare outcomes receive too much decision weight. When a rare outcome with a probability *p* is desirable, its impact (given by $w\left( p \right)$) is overweighed because of the overweighting of small probabilities ($w\left( p \right) > p$). This overweighting increases the attractiveness of the prospect concerned, leading to (probabilistic) risk-seeking behavior and the possibility effect. Similarly, when a rare outcome with a probability *p* is unfavorable, its impact (given by $1 - w\left( {1 - p} \right)$) is overweighted because of the underweighting of large probabilities ($w\left( {1 - p} \right) < 1 - p$). This overweighting decreases the attractiveness of the prospect concerned, leading to (probabilistic) risk aversion and the certainty effect.

The pattern of inverse S-shaped probability weighting is commonly interpreted as a reflection of both cognitive and motivational deviations from EU (Gonzalez and Wu 1999). On the one hand, the simultaneous overweighting and underweighting of extreme probabilities imply insufficient sensitivity to intermediate probabilities. This effect is called “likelihood insensitivity,” and it points to cognitive limitations in discriminating among different levels of uncertainty. On the other hand, an underweighting of moderate probabilities (such as $w\left( {0.5} \right) < 0.5$) suggests a pessimistic attitude toward risk across most of the probability domain. The presence of this effect points to motivational deviations from EU.

An alternative interpretation of inverse S-shaped probability weighting was given by Pachur et al. (2017). This interpretation is based on bounded rationality. Probability weighting can also reflect heuristic information processing: while likelihood insensitivity characterizes the propensity of a choice heuristic to use any information about probabilities in a decision process (e.g., in the priority heuristic proposed by Brandstätter et al. (2006)), pessimism and optimism characterize the use of maximum or minimum outcomes in assessing the prospects (e.g., in maxmin or maxmax heuristics).

## Relation to previous DFE literature

Hertwig and Erev (2009) considered three DFE paradigms: a partial feedback paradigm, a full feedback paradigm, and a sampling paradigm. The two feedback paradigms involved repeated choices, where the feedback was either about the realized outcome only (partial feedback; Barron and Erev 2003), or about both the realized and the foregone outcome (full feedback; Yechiam and Busemeyer 2006). Differently, the sampling paradigm involved a single (rather than repeated) choice, which was preceded by a purely exploratory and inconsequential sampling period, during which the subjects drew outcomes from unknown payoff distributions, usually with replacement (Hertwig et al. 2004; Weber et al. 2004). Hertwig and Erev (2009) noted that all three of these paradigms lead to a robust and systematic description-experience gap. As we investigate probability weighting under RDU in this study, we confine our attention to the sampling paradigm of DFE. Note that most economic models of choice under risk and uncertainty (including EU, RDU, and PT models) are designed to capture single decisions, and the above-mentioned evidence on probability weighting is almost exclusively based on decision tasks of this type. The subsequent subsections clarify the relation of our study to previous studies concerning the sampling paradigm.

### Autonomous sampling design versus regulated sampling design

In the original sampling paradigm of DFE as discussed by Hertwig et al. (2004), subjects have complete autonomy in their information searches. This autonomy means that every subject decides how many draws to make, when to stop sampling, and when to proceed to the choice stage by herself. The autonomous sampling design has crucial implications for the observed choice behavior in DFE experiments.

The first implication of this design concerns the sampling error. Under complete autonomy, subjects show a strong behavioral tendency to rely on small samples (insufficient information searches), which results in under- or non-observation of rare outcomes. Sampling error has been shown to be the primary source for the classic description-experience gap (Fox and Hadar 2006; Wulff et al. 2018). Reliance on small samples may also result in the overestimation of rare outcomes when a rare outcome is experienced in a small sample. For example, when considering a sample of five observations, a subject can experience relative frequencies only in increments of 0.2. Such an experience of rare outcomes leads to an overestimation of small probabilities (e.g., a probability of 5%) and amplification of the differences between options in terms of expected values. This so-called amplification effect has been shown to reduce the discriminability of probability weightings under DFE (Broomell and Bhatia 2014; Hertwig and Pleskac 2010; Hau et al. 2010).

**Fig. 2:**
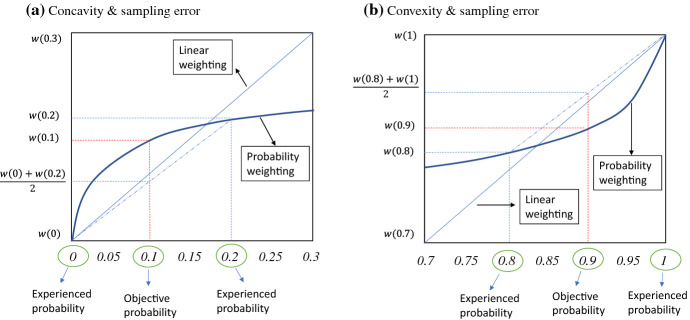
Distortions due to aggregation

The second implication of the autonomous sampling design concerns an aggregation problem that arises due to a lack of control over the individual sampling experiences. Each subject in an autonomous sampling design makes choices based on her own experienced probabilities. Notably, the aggregation of such individual choices amounts to taking the average of the weightings, rather than the weighting of the average, of the experienced probabilities. Consequently, the concave-convex curvature of the inverse S-shaped probability weighting function can lead to an erroneous description-experience gap. This problem is demonstrated in Fig. 2. To further illustrate, suppose that each subject involved in DFE draws only five times, with half of the subjects never experiencing a rare outcome, and the other half experiencing it once. This result gives experienced probabilities of either 0% or 20%. As Fig. 2a shows, aggregating the choices of all the subjects amounts to averaging the weightings of 0% and 20%, rather than weighting the average of 0% and 20%, which is 10%. Therefore, the aggregate choice appears to indicate that 10% is underweighted due to concavity. Figure 2b shows a dual effect, in which a convex probability weighting for large probabilities moves the aggregate choices in the direction of overweighting. Thus, together with the concavity for small probabilities, this pattern implies a reversed inverse S at the aggregate level.

Another implication of autonomous sampling concerning sampling with replacement is ignorance regarding the set of possible outcomes. Specifically, a subject who is unaware of the certainty or possibility of various prospective outcomes can never ensure, based on a finite number of observations, that an always-experienced outcome is actually certain, or that a never-experienced outcome is actually impossible, if the sampling is done with replacement. The condition of ignorance particularly poses a problem for consistent evaluations of prospects in terms of RDU, as the model requires a complete ranking of the possible outcomes. For example, a subjective belief that an always-experienced outcome (whose certainty is unknown to the subject) is less than certain might, in fact, result in a reversed certainty effect. Such impacts of ignorance have been empirically demonstrated by Hadar and Fox (2009) and Glöckner et al. (2016). Abdellaoui et al. (2011b) addressed this issue by providing subjects with descriptive information about possible outcomes in DFE.

To address the issues above, our CSP serves to regulate sampling experience by requiring complete sampling from finite outcome distributions without replacement. Hence, the CSP equates the subjects’ experienced probabilities (i.e., the observed relative frequencies) with the objective probabilities. Thus, the CSP not only controls for the sampling error^2^ but also facilitates the consistent evaluation of prospects under RDU.

Previous studies on DFE have also attempted to use different regulated sampling designs to control for sampling error. In a study by Hau et al. (2008), the subjects were required to draw large samples. In the study by Ungemach et al. (2009), the subjects drew samples that were accurate representations of the underlying outcome probabilities. In these studies, the sampling was done with replacement, unlike in the case of CSP. Therefore, no complete knowledge of the objective probabilities was attainable from the finite sampling experience.

### DFD versus DFE: the role of ambiguity

The previous evidence on probability weighting under DFE is rather mixed, possibly due to differences in sampling design, methodology, or in the types and levels of analysis. A detailed table on previous studies is presented in Online Appendix 4.

Some previous studies have also indicated that different attitudes toward known and unknown outcome probabilities (risk vs. ambiguity) are another source for the description-experience gap. By using a design that was intermediate between DFE and DFD, Abdellaoui et al. (2011b) documented ambiguity-induced pessimism (i.e., *ambiguity aversion*) and attenuated overweighting (rather than underweighting) of small probabilities under DFE. Similar findings were also documented in Kemel and Travers (2016) and Cubitt et al. (2019), whose experimental methodologies were comparable to that of Abdellaoui et al. (2011b). Using designs that were closer to the original sampling paradigm, Glöckner et al. (2016) and Kellen et al. (2016) reported even more pronounced inverse S-shaped probability weighting under DFE than under DFD, which is parallel to the *ambiguity*-*generated likelihood insensitivity* that has been commonly documented in the economics literature on ambiguity (Abdellaoui et al. 2011a; Dimmock et al. 2016; Fox and Tversky 1998; Tversky and Fox 1995; Wakker 2004).

The CSP, by design, represents a case of risk, as the objective probabilities are available to the subjects through sampling experience.^3^ Among the previous studies, only the study by Barron and Ursino (2013) investigated the description-experience gap under risk (their experiment 1). However, their study made inferences only about the relative weightings of rare outcomes under DFE compared with those under DFD but not about the actual over- or under-weighting of rare outcomes under DFE.

### The description-experience gap

Our study is primarily concerned with investigating the gap between the complete sampling and description treatments in probability weighting under the RDU framework. It is important to clarify that the description-experience gap originally introduced by Barron and Erev (2003) and Hertwig et al. (2004) mainly referred to changes in choice propensities, rather than to measurements of probability weighting functions or other components of PT or RDU. Hertwig and Erev (2009) wrote that “underweighting of rare events as measured in terms of the parameters of the decision-weighting function of cumulative prospect theory is not a necessary condition for the description-experience gap” (p. 521). Barron and Erev (2003), Hertwig et al. (2004) and Hertwig et al. (2006) explained the choice gap as a product of reliance on small samples and the recency effect generated by an adaptive learning process. In addition, the implications that the description-experience gap may have for probability weighting functions have been another topic of interest, mostly among researchers in economics. This aspect of the problem has also been the subject of recent research on DFE, as mentioned in the preceding subsection.

Although the gap in probability weighting is probably the most well-known one, similar gaps between description and experience have also been documented in other behavioral phenomena. Erev et al. (2017) indicated discrepancies in 14 different behavioral phenomena (including reflection effect and loss aversion as captured by PT) in situations where the subjects made repeated decisions with access to both feedback and descriptions of prospects. Ert and Trautmann (2014) indicated a reversal of attitudes toward ambiguity, i.e., changes in preferences between risky and ambiguous prospects, which could arise due to sampling experience. Ert and Haruvy (2017) found a convergence toward risk neutrality by using the risk aversion measure in Holt and Laury (2002), assuming EU in a situation where the subjects made repeated decisions with feedback.

## Method

Our experimental procedure involved two stages. In the first stage, the utility function of each subject was elicited by using the trade-off (TO) method proposed by Wakker and Deneffe (1996). The TO method is a well-established technique that is commonly used in studies that investigate probability weighting (Abdellaoui 2000; Abdellaoui et al. 2005, 2007; Bleichrodt and Pinto 2000; Etchart-Vincent 2004, 2009; Qiu and Steiger 2010). This method involves eliciting a standard sequence of outcomes that are equally spaced in utility units. The elicitation procedure consists of a series of adaptive indifference relations. For two fixed outcomes, *G* and *g*, and a selected starting outcome $x_{0}$ with $x_{0} > G > g, \, x_{1} > x_{0}$ is elicited such that the subject is indifferent between the prospects $x_{1_{p}}g$ and $x_{0_{p}}G$. Then, $x_{1}$ is used as an input to elicit $x_{2} > x_{1}$ such that the subject is indifferent between $x_{2_{p}} g$ and $x_{1_{p}} G$. This procedure is repeated *n* times to obtain the standard sequence $\left( {x_{1} , \ldots , x_{n} } \right)$ with indifferences $x_{i + 1_{p}} g\sim x_{i_{p}} G$ for $0 \le i \le n - 1$. Under RDU, these indifferences result in $U\left( {x_{1} } \right) - U\left( {x_{0} } \right) = U\left( {x_{2} } \right) - U\left( {x_{1} } \right) = \ldots = U\left( {x_{n - 1} } \right) - U\left( {x_{n} } \right)$ (for the derivation, see “Appendix A”). One remarkable feature of the TO method is that it elicits these equalities irrespective of what the probability weighting is. Therefore, this method is robust against most distortions due to non-expected utility maximization.

We used parametric estimation of utilities, rather than linear interpolation, to smooth out errors, and to better capture the utility curvature. We also used power utility, which has been widely favored in previous empirical tests reported in the literature (Stott 2006; Camerer and Ho 1994). Once the standard sequence of outcomes had been obtained, we acquired the utility function of each individual by parametrically estimating the power specification $U\left( x \right) = x^{\alpha }$ with $\alpha > 0$. after scaling of $x_{i}$ as $x_{i} = \frac{{x_{i} - x_{0} }}{{x_{n} - x_{0} }}$. The parameter $\alpha$ was calculated by using an ordinary least squares regression without intercept, ${\text{log}}\left( {U\left( x \right)} \right) = \alpha \log \left( x \right) + \varepsilon$ where $\varepsilon \sim N\left( {0,\sigma^{2} } \right)$.

In the second stage of our procedure, we measured probability weighting using several binary choice questions. These questions were constructed on the basis of the subject-specific outcome sequences obtained from the first stage. The subjects chose between a risky prospect $x_{k_{q}} x_{j}$ and a sure outcome $s_{q}$, where $x_{k}$ and $x_{j}$ were two distinct elements of the elicited outcome sequence with $x_{k} > x_{j}$, and where $s_{q}$ was equal to the certainty equivalent of $x_{k_{q}} x_{j}$ under EU.

1 $$s_{q} = U^{ - 1} \left[ {qU\left( {x_{k} } \right) + \left( {1 - q} \right)U\left( {x_{j} } \right)} \right].$$

This means that $s_{q}$ would be equivalent to $x_{k_{q}} x_{j}$ if the subject with the given utility did not weigh probabilities. Hence by construction, the following logical equivalences held for the given preference relations under RDU.

2 $$x_{k_{q}} x_{j} \prec s_{q} \Leftrightarrow w\left( q \right) < q \, \left({underweighting } \right)$$

3 $$x_{k_{q}} x_{j} \sim s_{q} \Leftrightarrow w\left( q \right) = q \, \left( {EU} \right)$$

4 $$x_{k_{q}} x_{j} \succ s_{q} \Leftrightarrow w\left( q \right) > q \, \left( {overweighting} \right)$$

As we did not allow indifference in our experiment, each choice revealed either the overweighting or underweighting of probability *q*. Our method made the deviations from EU observable at the aggregate level. For instance, an overweighting of *q* could be detected when the majority of subjects choose the risky $x_{k_{q}} x_{j}$, as in logical equivalence (4).

## The experiment

### Subjects and incentives

The experiment was performed at the ESE-EconLab at Erasmus University in five group sessions. The subjects were 89 Erasmus University students from various academic disciplines (average age 23 years, 40 females). All of the subjects were recruited from a pool of subjects who had never before participated in any economics experiment in our lab, as we sought to avoid subjects who had experienced the TO method. We paid each subject a €5 participation fee. Besides, at the end of each session, we randomly selected two subjects who could play out one of their randomly drawn choices for real. The ten subjects who played for real received €60.70 on average. Over the whole experiment, the average payment per subject was €12.37.

### Procedure

The experiment was run on computers. The subjects were separated by wooden panels to minimize interaction. All of the subjects were provided with paper and pen, and they were instructed that they could take notes if they wished to. Taking notes was not obligatory. Before starting the experiment, the subjects read the general instructions, which included detailed information on the payment procedure, the user interface, and the types of questions they would face. They were allowed to ask questions at any time during the experiment. The experiment consisted of two successive stages without a break in between. Each stage started with a set of instructions and several training questions to familiarize the subjects with the stimuli. These experimental instructions are given in full in Online Appendix 1. Each session took 45 min on average, including the payment phase after the experiment.

### Stimuli

#### Stage 1: measuring utility

In the first stage of the experiment, a standard sequence of outcomes was elicited by using the TO method. We measured $x_{1} ,x_{2} ,x_{3} ,x_{4}$, and $x_{5}$ from the following five indifferences, with $p = 0.33,\, G = 17,\, g = 9,$ and $x_{0} = 24$:

$$24_{p}G \sim x_{1_{p}}g, \ x_{1_{p}}G \sim x_{2_{p}}g,\; x_{2_{p}} G \sim x_{3_{p}}g,\; x_{3_{p}}G \sim x_{4_{p}}g,\;{\text{and}}\;x_{4_p}G \sim x_{5_p}g.$$

**Fig. 3:**
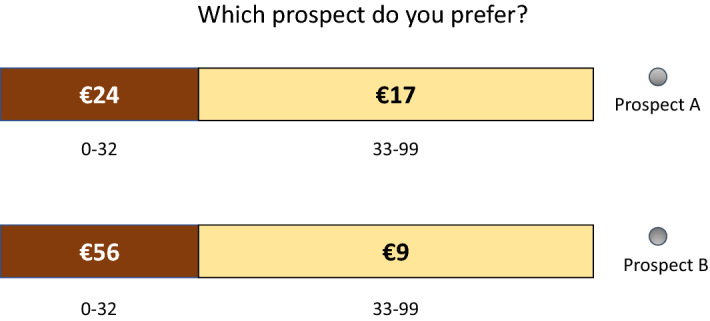
The choice situation in the TO part

The indifferences were obtained through a bisection method that required seven iterations for each $x_{i}$. In addition, the last iteration of one randomly chosen $x_{i}$ was repeated at the end of stage 1, to test the reliability of the indifferences. Hence, the subjects answered a total of 36 questions in this part of the experiment. The bisection iteration procedure is described in “Appendix B”. The prospects were presented on the screen, as illustrated in Fig. 3.

In this part of the experiment, risk was generated by two ten-faced dice, with each die generating one digit of a random number from 00 to 99. In cases where a choice question from this part was implemented for real at the end of the experiment, the outcome of each prospect depended on the result of two dice physically rolled by the subjects.

#### Stage 2: description versus sampling

Before the start of the experiment’s second part, each subject was randomly assigned to one of the two treatments: description or complete sampling. Here and throughout the next section, we refer to the latter in short as “sampling treatment.” In both treatments, the subjects had to answer seven subject-specific binary choice questions. Each question entailed a choice between a risky prospect $x_{5_{q}} x_{1}$ and the safe prospect $s_{q}$, as further described in the method section. Note that both $x_{1}$ and $x_{5}$ were endogenously determined and varied between subjects.^4^ The values of $s_{q}$ were always rounded to the nearest integer. The seven probabilities used for the investigation of probability weighting were $0.05, 0.10, 0.20, 0.50, 0.80, 0.90\;{\text{and}}\;0.95$. Within each treatment, the orders of the seven questions were counterbalanced. The position of the risky prospect and the safe prospect were also randomized in each question.

**Fig. 4:**
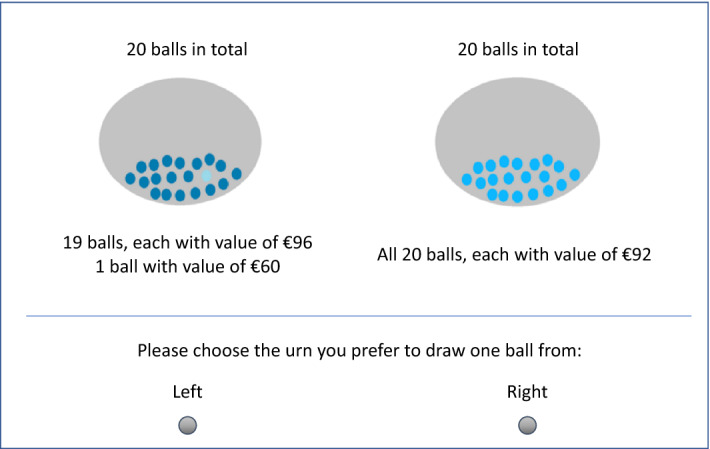
A choice situation in the description treatment

The prospects were represented by Ellsberg-type urns, each containing 20 balls with various monetary values attached to them. This way, all the aforementioned probabilities were fractions of 20; i.e., 5% was 1 out of 20, 10% was 2 out of 20, etc. The two treatments differed in terms of how the subjects learned the contents of the urns. In the description treatment, the contents of the urns were explicitly described to the subjects. Figure 4 shows a screenshot of a choice situation for the description treatment.

**Fig. 5:**
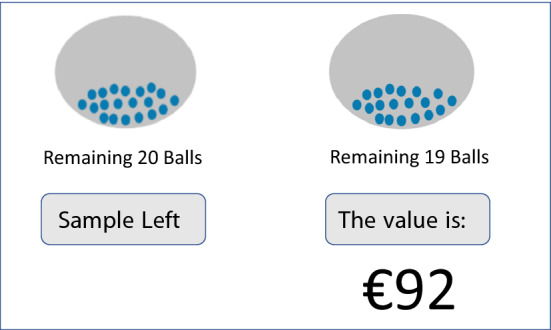
Sampling stage in the sampling treatment

Subjects in the sampling treatment were initially given no information about the contents of the urns except the total number of balls. They could only learn about the content of the urns by sampling each ball one-by-one without replacement, and observing the monetary values attached. Figure 5 shows a screenshot of the sampling phase in the sampling treatment. The subjects sampled balls from the urns by clicking “Sample left” or “Sample right” on the screen. Each time they made a selection, the monetary outcome attached to the ball sampled was shown to the subject for 1.5 s before the message disappeared. The subjects could sample the balls at their speed, in whichever order they preferred, and could switch as many times as they wanted, but they could only proceed to the choice stage after sampling all of the balls in both urns.

**Fig. 6:**
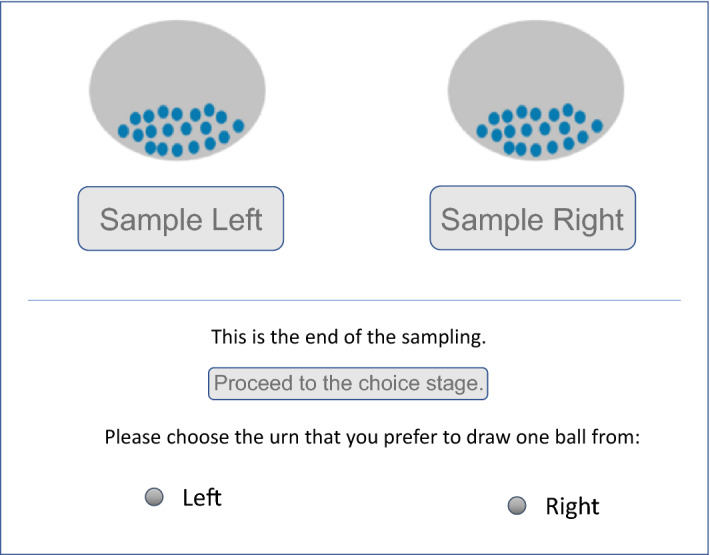
Choice stage in the sampling treatment

Figure 6 shows a screenshot of the choice stage in the sampling treatment. In case a question in this part was implemented for payment at the end, the experimenters set up physical, opaque urns (similar to those that presented on the screen). Each urn was filled with 20 ping-pong balls that were painted either dark blue or light blue, with these colors being associated with the payoffs in question (see Fig. 4). The subjects physically drew a ball from the urn, which determined their payoffs.

The subjects in the description treatment had to answer 21 additional questions following the primary set of 7 questions, to equalize the length of the two treatments. These additional questions concerned another research project.

## Results

### Reliability and consistency of utility elicitation

In the TO part of the experiment, each subject repeated one choice that she had faced in one of the five indifference elicitations. The repeated choice was randomly selected among the last steps of the iterations. As the subjects were very close to indifference at the last step, this choice was the strongest test of consistency. The subjects made the same choices that they made previously in 70.8% of the cases. Reversal rates up to one third are common in the literature (Stott 2006; Wakker et al. 1994). Especially if the closeness to indifference is considered, the reversal rates we found were satisfactory. Among the reversed cases, repeated indifferences were higher than the original indifference values in 42.3% of the cases, which did not suggest a systematic pattern ($p = 0.56$, two-sided binomial). Overall, the repeated indifference values did not differ from those of the original elicitations ($p = 0.44$, Wilcoxon sign-rank).

### Utility functions

Table 1 gives descriptive statistics for the elicited outcome sequence.^5^ The parameter $\alpha$ of the power utility $u\left( x \right) = x^{\alpha }$ was estimated at the individual level by ordinary least squares regression. The average $R^{2}$ was 0.985, which indicated that our estimations fit the data very well.^6^

**Table 1:** Descriptive statistics for the elicited outcome sequence **(***N = *88)

	Mean	SD	Min	Median	Max
$x_{0}$	24.00	0.00	24.00	24.00	24.00
$x_{1}$	60.36	23.48	30.00	58.00	118.00
$x_{2}$	90.36	42.58	36.00	80.00	212.00
$x_{3}$	125.23	65.89	46.00	102.00	306.00
$x_{4}$	164.18	91.13	52.00	134.00	400.00
$x_{5}$	204.14	116.25	58.00	160.00	494.00
$\alpha$	1.05	0.36	0.41	0.99	2.65

The summary statistics for the mean and median α are reported in the last row of Table 1. The aggregate data did not deviate from linearity ($p = 0.92$, Wilcoxon sign-rank). Although the mean $\alpha$ suggested slight convexity, this result was affected by the outliers in our data. Three subjects exhibited extreme convexity with $\alpha > 2$, and the skewness/kurtosis test rejected the normality of the distribution of $\alpha '$ s ($p < 0.01$). The utility estimations did not differ across the two treatments ($p = 0.84$, Wilcoxon rank-sum).^7^

Our data suggested slightly more evidence for concavity than for convexity at the individual level. Considering those subjects whose $\alpha$ parameters were significantly different from 1 (at a 5% significance level), we find that 30 subjects (15 in the sampling treatment and 15 in the description treatment) exhibited concavity ($\alpha < 1$), and 23 subjects (12 in the sampling treatment and 11 in the description treatment) exhibited convexity ($\alpha > 1$). The proportions of concave and convex utilities did not differ ($p = 0.41$, two-sided binomial). The remaining 35 subjects (40%) did not exhibit significant deviations from linear utility.

### Probability weighting: description versus sampling

#### Aggregate data

**Fig. 7:**
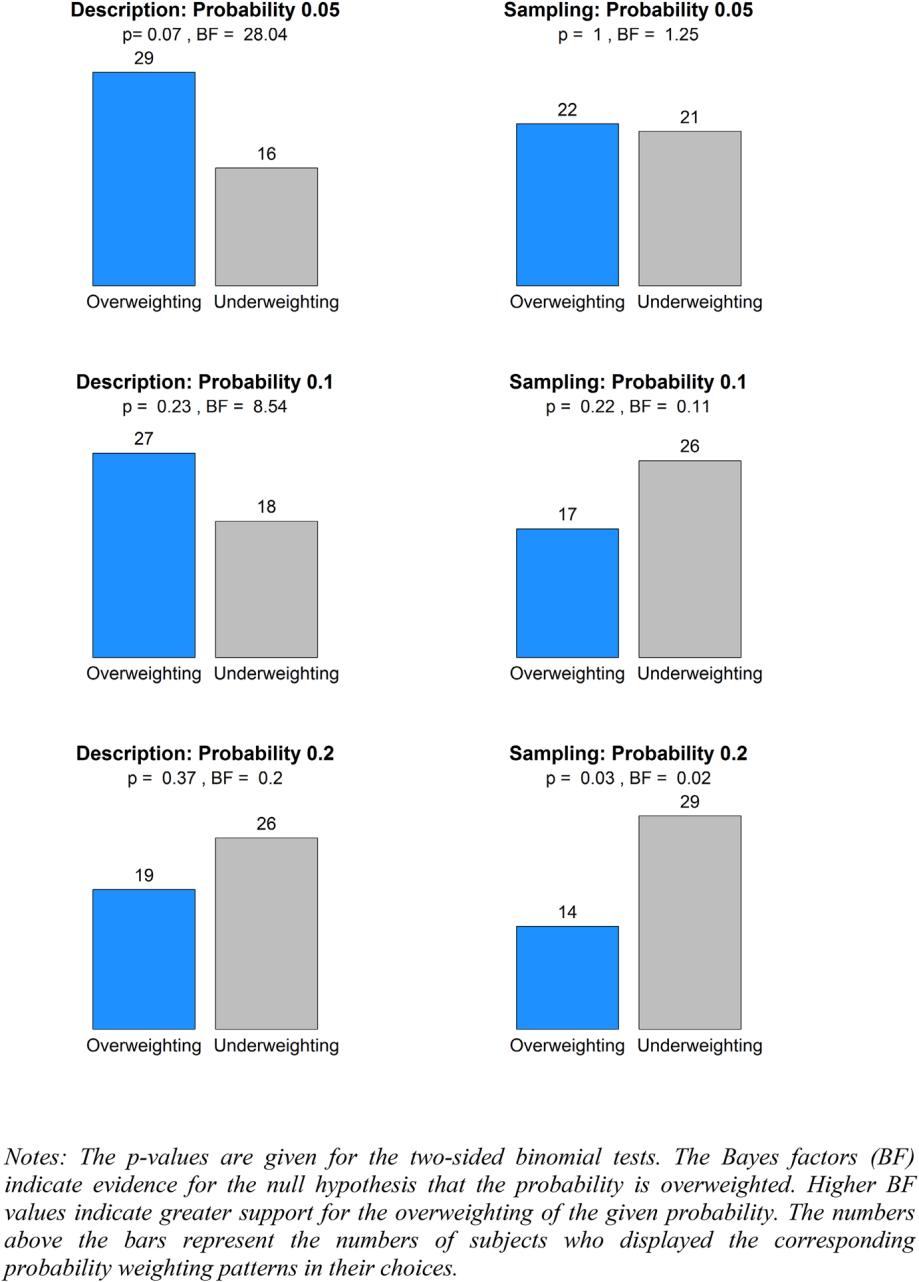
The weighting of small probabilities

**Fig. 8:**
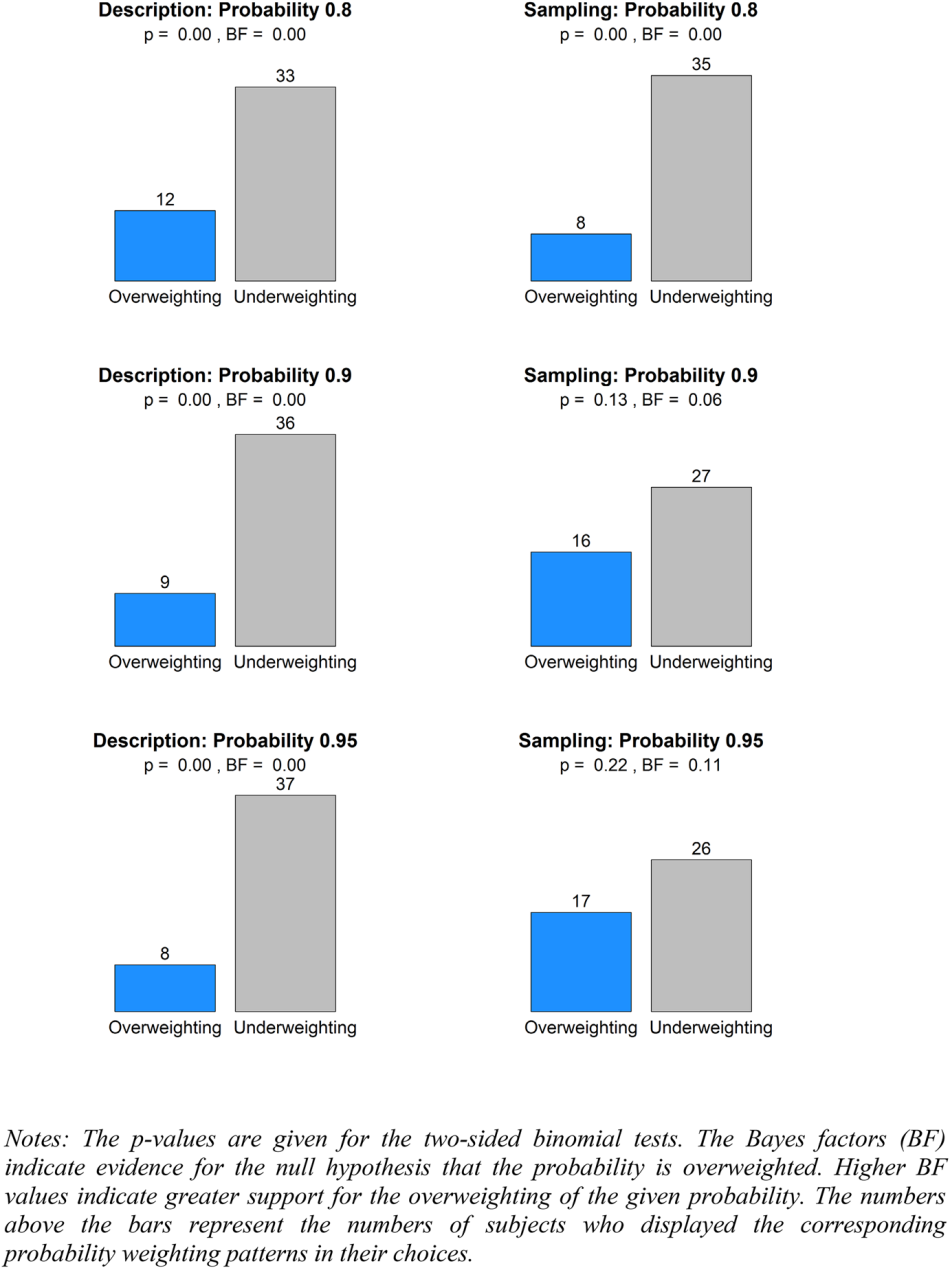
The weighting of large probabilities

In this section, we report the aggregate choices in the direction of overweighting and underweighting according to logical equivalences (2) and (4) (as presented in the “Method” section). The proportions of overweighting and underweighting of small and large probabilities are given in Figs. 7 and 8, respectively.

The aggregate choices replicated the common description-experience gap at the extreme probabilities. Overall, the observed gap indicated significantly less overweighting of rare outcomes under the sampling treatment, when assessed on the basis of a repeated-measures logistic regression ($z = - 2.15,p = 0.031$).^8^ Based on individual hypothesis tests, the gap was significant at 0.95 ($p = 0.02, \chi^{2}$); and was marginally significant at 0.10 and 0.90 ($p = 0.06$, and $p = 0.07$ respectively, $\chi^{2}$). The gap at probability 0.05 was not significant ($p = 0.20,\;\chi^{2}$), although the trend suggested reduced overweighting in the sampling treatment. Also, no description-experience gap was apparent in the middle range, $0.20 \le q \le 0.80$ ($p = 0.35$, $p = 0.92$, and $p = 0.37$ for $q = 0.20, 0.50$, and 0.80 respectively, $\chi^{2}$).

In what follows, we focus on the absolute overweighting and underweighting of probabilities under the two treatments. We first test the deviations from unbiased weighting in either direction by using the two-sided binomial tests for proportions. In addition, to interpret the relative evidence for overweighting and underweighting, we report the Bayes factors for the null hypothesis of overweighting against the alternative hypothesis of underweighting. The Bayes factors indicate the relative evidence for the null hypothesis. For instance, a Bayes factor of 10 indicates that overweighting is 10 times more likely than underweighting for the given probability. Following Jeffreys (1961), we interpret a Bayes factor between 3 and 10 as “some evidence,” a Bayes factor between 10 and 30 as “strong evidence,” and a Bayes factor larger than 30 as “very strong evidence” for the null hypothesis of overweighting. Similarly, Bayes factors of between 0.1 and 0.33, between 0.03 and 0.1, and less than 0.03 are interpreted as “some evidence,” “strong evidence,” and “very strong evidence,” respectively, for the alternative hypothesis of underweighting.^9^

As shown in Fig. 7, for the small probabilities under the description treatment, we found a marginally significant deviation from unbiased weighting at the probability of 0.05 ($p = 0.07$). Interpreting the results in terms of Bayes factors, we found strong evidence of overweighting 0.05 ($BF = 28.04$), some evidence of overweighting 0.1 ($BF = 8.54$) and some evidence of underweighting 0.2 ($BF = 0.2$). Turning to the small probabilities under the sampling treatment, we found a significantly biased weighting only at the probability of 0.2 ($p = 0.03$). Interpreting the results in terms of Bayes factors, we found strong evidence of underweighting 0.2 ($BF = 0.02$) and some evidence of underweighting 0.1 ($BF = 0.11$). We found almost no evidence for the underweighting or the overweighting of 0.05 ($BF = 1.25$).

For the large probabilities (as shown in Fig. 8) under the description treatment, we found significant biases in the weighting of probabilities 0.8, 0.9, and 0.95 ($p < 0.01$ for all). The Bayes factors indicated very strong evidence for underweighting of 0.8, 0.9 and 0.95 ($BF < 0.03$ for all). Under the sampling treatment, we found significant bias only at 0.8 ($p < 0.01$). The Bayes factors suggested very strong evidence of underweighting 0.8 ($BF < 0.03$), strong evidence of underweighting 0.9 ($BF = 0.06$), and some evidence of underweighting 0.95 ($BF = 0.11$).

Last, we examined the weighting of the moderate 0.5 probability. In the description treatment, 38 out of 45 subjects underweighted 0.5. In the sampling treatment, 36 out of 43 subjects underweighted 0.5. Hence, the deviations from unbiased weighting were highly significant at 0.5 in both treatments ($p < 0.01$ for both treatments, two-sided binomial tests). The Bayes factors also indicated very strong evidence in favor of under-weighting at 0.5 ($BF < 0.03$ for both treatments).

To summarize, our aggregate data replicated the commonly observed inverse S pattern under the description treatment, but provided no evidence for a reversal of the inverse S pattern under the sampling treatment. In particular, we did not observe significant deviations from unbiased weighting at the extreme probabilities 0.05, 0.1, 0.9, or 0.95 in cases where the objective probabilities were learned from sampling without replacement. Notably, no convincing evidence was found for the underweighting of small probabilities 0.05 and 0.1, and more evidence was found for the underweighting than for the overweighting of large probabilities.

#### Individual data

Next, we examined the shapes of the probability weighting functions at the individual level. We classified each subject’s probability weighting function as inverse S-shaped, S-shaped, pessimistic, or optimistic, according to the numbers of over- and under- weightings of three small and three large probabilities, as illustrated in Figs. 7 and 8. These four classes of the probability weighting functions are exhaustive. Specifically, a probability weighting function is inverse S-shaped if it simultaneously overweights at least two out of three small probabilities and underweights at least two out of three large probabilities. The opposite pattern implies an S-shaped probability weighting function. Similarly, a pessimistic probability weighting function underweights at least two small and two large probabilities at the same time, and the opposite pattern implies an optimistic probability weighting function.

Table 2 shows the results of this classification. The probability weighting functions were mainly classified as inverse S-shaped, S-shaped, or pessimistic, and the proportion of optimistic weighting functions was negligible in both treatments. Among the three main types of the probability weighting functions, the majority of cases in the description treatment were inverse S-shaped ($p < 0.01$, one-sided binomial, H0: The proportion of inverse S is $\frac{1}{3}$ among inverse S, S, and pessimistic types). Among participants in the sampling treatment, the inverse S-shape was also the most frequently observed, but it did not constitute the majority of cases ($p = 0.13$, one-sided binomial, H0: Proportion of inverse S is $\frac{1}{3}$ among the inverse S, S, and pessimistic types).

**Table 2:** Types of probability weighting functions

	Inverse S-shaped	S-Shaped	Pessimistic	Optimistic
Description	51% (23)	9% (4)	36% (16)	4% (2)
Sampling	42% (18)	23% (10)	33% (14)	2% (1)
Gap	9p.p. (*p* = 0.40)	− 14p.p. (*p* = 0.08)	3p.p. (*p* = 0.82)	2p.p. (*p* = 1)

The numbers of probability weighting functions are given in the parentheses. The p-values are results from the (two-sided) Fisher’s exact test

A comparison across the two treatments indicated that the proportion of S-shaped probability weighting functions was higher in the sampling treatment, although the difference was only marginally significant ($p = 0.08$, two-sided Fisher’s exact test). No significant difference appeared between the proportions of inverse S-shaped, pessimistic, and optimistic probability weighting functions across the two treatments.

Overall, our individual-level analysis suggested a reduced but persistent inverse S pattern in the sampling treatment. The results reported above are valid without requiring any parametric assumptions regarding probability weighting or specifications on the stochastic nature of errors. The parametric analysis in the next section supplements our nonparametric results.

#### Parametric estimations

We performed our parametric analysis of the probability weighting functions by implementing a Bayesian hierarchical estimation procedure. This procedure enables reliable aggregate and individual-level estimations with limited data available per subject. The procedure was recommended by Nilsson et al. (2011) and Scheibehenne and Pachur (2015). It has been applied in several other studies for estimating RDU and PT components (Balcombe and Fraser 2015; Kellen et al. 2016; Lejarraga et al. 2016).

We estimated the Goldstein and Einhorn (1987) weighting function, as given by $w\left( q \right) = \frac{{\delta q^{\gamma } }}{{\delta q^{\gamma } + \left( {1 - q} \right)^{\gamma } }}$.^10^ The parameter $\gamma$ determines the curvature and captures the sensitivity toward changes in probabilities. In this function, $\gamma < 1$ indicates an inverse S-shape and likelihood insensitivity, and $\gamma > 1$ indicates S-shape and likelihood oversensitivity. The parameter $\delta$ determines the elevation and captures the degree of pessimism. For $\delta = 1,$ we have $w\left( {0.5} \right) = 0.5$. Lower (higher) values of $\delta$ indicate less (more) elevation and more (less) pessimism. Following Kruschke (2011), we evaluated the credibility of likelihood insensitivity and pessimism based on the ranges of 95% intervals from the posterior distribution of parameters. The details on estimation procedures are given in Online Appendix 5.

**Fig. 9:**
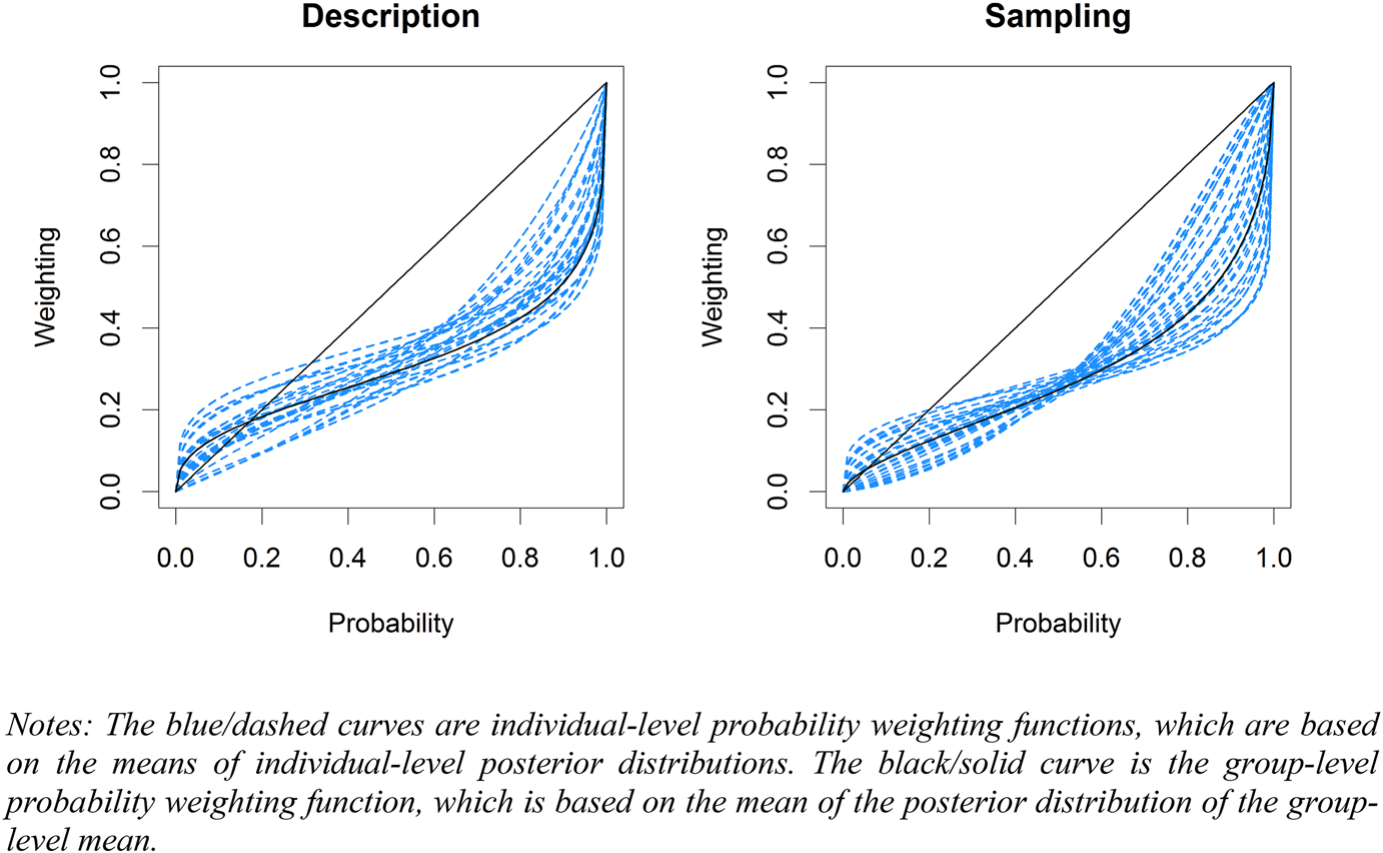
Probability weighting functions

We report the estimated group-level mean parameters and the corresponding 95% credibility intervals in Table 3. Figure 9 shows the estimated probability weighting functions. The estimated parameters indicated credible likelihood insensitivity and pessimism in both treatments, as $\gamma = 1$ and $\delta = 1$ both fell on the right side of the 95% credibility intervals. The description-experience gap in terms of likelihood insensitivity and pessimism was not found to be credible, although the difference in likelihood insensitivity was suggestive. Hence, we observed a less pronounced inverse S-shaped weighting function in the sampling treatment, although the elevation was comparable across the two treatments (see the solid curves in Fig. 9).

**Table 3:** Group level mean parameters

	$\gamma$	$\delta$
Description	0.430 [0.234, 0.675]	0.407 [0.259, 0.590]
Sampling	0.611 [0.372, 0.868]	0.331 [0.198, 0.508]
Gap	− 0.181 [− 0.517, 0.160]	0.076 [− 0.152, 0.304]

The estimated parameters are the means of the posterior distributions of the group level means. 95% credibility intervals are given in square brackets

At the individual level, pessimism ($\delta < 1$) was credible for all of the subjects in both treatments. Likelihood insensitivity was credible for 51% (23 out of 45) of the subjects in the description treatment and for 29% (13 out of 43) of the subjects in the sampling treatment. Although there was no subject with likelihood oversensitivity ($\gamma > 1$) in the description treatment, 23% (10 out of 43) of the subjects in the sampling treatment exhibited likelihood over-sensitivity, although it was never credible. These results confirmed our previous nonparametric results at the individual level.

## Discussion

### The two-stage methodology

Our experiment used a two-stage design that separates the measurement of utilities from the measurement of probability weighting. This design circumvented the identification problems that can be caused by potential interactions (collinearities) between utility and probability weighting in simultaneous parametric estimations (Gonzalez and Wu 1999, pp. 152; Scheibehenne and Pachur 2015, pp. 403–404; Stott 2006, pp. 112; Zeisberger et al. 2012). Our parametric Bayesian hierarchical estimations avoided further averaging biases due to heterogeneous preferences (Nilsson et al. 2011; Regenwetter and Robinson 2017). To test the descriptive adequacy of our Bayesian estimations, we compared posterior predictions of the estimated model with the actual data observed (see Online Appendix 5, Figure A5.2). We found that the model was accurate in predicting choices.

One may still be concerned about potential interdependencies between the utility and probability weighting measurements in our two-stage design. Our measurement of probability weighting in the second stage depended on the utilities elicited in the first stage. Thus, any error in the calculation of $s_{q}$ from the first stage could have resulted in a bias in probability weighting measurements. To control for this kind of error propagation, we tested the internal validity of the utility measurements through consistency checks on the elicitations of standard sequences. We used the most stringent test for consistency (see Sect. 5.1 and “Appendix B”), and we found that the rate of consistency was high. In addition, we used parametric fitting in our utility estimations to smooth out the errors (Bleichrodt et al. 2010; Etchart-Vincent 2004). We observed high goodness-of-fit in our estimations of utility. The direction of rounding in the calculation of $s_{q}$ values did not predict the choices in the second stage (see Online Appendix 7). Our utility estimations indicated slight utility curvature, with some heterogeneity at the individual level. These estimations were close to those reported in previous studies (Abdellaoui 2000; Abdellaoui et al. 2005; Bleichrodt et al. 2010; Qiu and Steiger 2010; Schunk and Betsch 2006). Our results replicated previous findings on the common inverse S pattern under DFD conditions, and we found the classic description-experience gap, which confirmed the validity of our design.

Another potential concern is the incentive compatibility of the TO method, due to its adaptive nature (with previous choices determine the later stimuli). No previous studies have found this compatibility issue to be a problem in practice (Abdellaoui 2000; Bleichrodt et al. 2010; Qiu and Steiger 2010; Schunk and Betsch 2006; van de Kuilen and Wakker 2011). In the terminology used by Bardsley et al. (2010), this issue might be a concern regarding theoretical incentive compatibility, but not a concern regarding behavioral incentive compatibility (p. 265). Still, as a precautionary measure, we included filler questions in the iteration process of our bisection procedure, intending to make the detection of the adaptive design even more difficult. Our data showed no evidence of strategic choices (see “Appendix B”).

### Beyond the information symmetry

The choice to use a CSP design was motivated by our desire to resolve the information asymmetry between the sampling and the description treatments. The information at the subjects’ disposal was equal in both treatments. However, our complete reliance on sampling experience (without any descriptive information about probabilities) still left room for a discrepancy between the information provided during the sampling stage and the information acquired, or utilized, by the subjects while they made their decisions. This feature of the CSP, which is also a crucial feature of the original paradigm of DFE, distinguishes the CSP from the DFD condition. An exploratory examination of the notes that were taken by the subjects during the experiment suggested the existence of different ways for processing sampling experience (see Online Appendix 8). In particular, although some subjects preferred to take very comprehensive notes of all their sampling observations, others noted only their sampled outcomes without mentioning their frequency or else took no notes at all.^11^ Such heterogeneity in mental processes possibly contributed to the gap observed between the two treatments in our study.^12^ For more discussion on the psychological factors involved in the gap, see Camilleri and Newell (2009).

### Experimental economics research on experience

This study highlights the economic relevance of the DFE research, which to date, has mostly been conducted by researchers in psychology. Our study re-examines the description-experience gap from a behavioral economic perspective. Our DFE experiment relates to the strand of economics literature that investigates the violations of the rationality benchmarks in economic theory. This strand of literature has generally claimed that the standard economic theory performs reasonably well in situations where there is sufficient opportunity for reflection on incentives, deliberation, learning, and experience (Plott 1996; Binmore 1999). Accordingly, extensive experimental studies have tested the impact of different types of experience on the common anomalies observed in choice experiments. Some examples from this literature include Loomes et al. (2003), who documented reductions in discrepancies between willingness to pay and willingness to accept due to repeated market experience. Other examples include Baillon et al. (2016) and Charness et al. (2007), who reported reductions in violations of stochastic dominance due to group deliberation and social interactions. van de Kuilen and Wakker (2006) and van de Kuilen (2009) reported significant convergence to EU maximization under risk in repeated choice settings in cases where immediate feedback was available. Humphrey (2006) reported reductions in violations of the independence axiom after observing resolutions of risky lotteries (see Bardsley et al. (2010) for further discussion of this literature).

Our experimental findings are mostly in line with the previous claims of the literature regarding reductions of irrationalities through experience and deliberation. Both the nonparametric and the parametric analyses indicate that the observed biases in probability weighting were reduced when the objective probabilities were learned more intuitively. Specifically, the sampling experience reduced both the certainty and the possibility effects. We would like to stress that although our study found no pattern of reversed inverse S-shaped probability weighting, this absence of evidence does not necessarily refute any previous assertions in the DFE literature. As clarified in Sect. 2.3, the original claims of underweighting for small probabilities refer to choice propensities, but not to measurements of probability weightings under RDU or PT. What our findings show is that the gap in choice propensities does not translate into a reversal of probabilistic risk attitudes under the RDU framework.

We hope that our study will further contribute to the economics literature by stimulating investigations of the various paradigms of DFE. Further studies involving DFE paradigms could be beneficial for economics research. First, DFD and DFE represent different real-life situations. Although some choice environments provide ample opportunities for learning from experience, others do not. For example, whereas repeated small-scale transactions in the market can allow for trial and error, decision-makers mainly rely on descriptions of the options in making more significant decisions, such as choosing a retirement or health plan. Understanding when and why people exhibit decision biases is ultimately informative for economic policymaking, and for possible implementations of the nudges (Thaler and Sunstein 2008).

Moreover, DFE is a rich experimental environment that can give rise to new theoretical approaches that provide alternatives to the Bayesian approach with EU. For example, some vital aspects of the sampling paradigm, such as memory, adaptive learning, and information search have been previously studied in the DFE literature (Ashby and Rakow 2014; Hertwig and Pleskac 2010; Hills and Hertwig 2010; Lejarraga et al. 2012; Kopsacheilis 2017; Ert and Haruvy 2017; Golan and Ert 2015). However, these aspects of sampling are not usually modeled in the traditional decision theories used in economics. To our knowledge, the only decision theory in economics that considers those related aspects is case-based decision theory, as proposed by Gilboa and Schmeidler (1995, 2001). Some of the empirical works that investigate this theory include those by Bleichrodt et al. (2017), Ossadnik et al. (2013) and Grosskopf et al. (2015).

## Conclusion

This study reconsiders the description-experience gap, which to date, has been mostly studied in the literature of psychology. We address the empirical question concerning the gap in risk attitudes induced by the non-linear weighting of probabilities. Our experimental findings support the existence of a description-experience gap, even in cases where objective probabilities from a finite number of sampling observations are available. However, we also find that this gap does not amount to a reversal of the inverse S-shaped probability weighting. In cases where decision-makers are allowed to learn about precise probabilities from experience, their sampling experience tends to reduce the cognitive impairment of likelihood insensitivity.

## Appendix A: Derivation of the standard sequence of outcomes in TO method

Under RDU, indifferences $x_{i + 1_{p}} g \sim x_{i_{p}} G$ imply $w\left( p \right)U\left( {x_{i + 1} } \right) + \left( {1 - w\left( p \right)} \right)U\left( g \right) = w\left( p \right)U\left( {x_{i} } \right) + \left( {1 - w\left( p \right)} \right)U\left( G \right)$. A rearrangement of this equation gives $U\left( {x_{i + 1} } \right) - U\left( {x_{i} } \right) = \frac{{\left( {1 - w\left( p \right)} \right)}}{w\left( p \right)}\left[ {U\left( G \right) - U\left( g \right)} \right]$ for all $0 \le i \le n - 1$. As the right-hand side of the equation is fixed by design, the indifferences result in $U\left( {x_{1} } \right) - U\left( {x_{0} } \right) = U\left( {x_{2} } \right) - U\left( {x_{1} } \right) = \ldots = U\left( {x_{n} } \right) - U\left( {x_{n - 1} } \right)$.

## Appendix B: Bisection procedure

The iteration process serves to measure $x_{1} ,x_{2} ,x_{3} ,x_{4} ,$ and $x_{5}$ on the basis of the following indifferences, in which $p = 0.33, G = 17, g = 9, x_{0} = 24:$

$$x_{0_{p}} G\sim x_{1_{p}} g,x_{1_{p}} G \sim x_{2_{p}} g,x_{2_{p}} G \sim x_{3_{p}} g,x_{3_{p}} G \sim x_{4_{p}} g, x_{4_{p}} G \sim x_{5_{p}} g$$

For each $x_{i}$, it took five choices to reach the indifference point. Subjects always chose between two prospects: $x_{i_{p}} g$ and $x_{i - 1_{p}} G$ for $i = 1, \ldots ,5$. The procedure was as follows.

1. The initial value of $x_{i}$ was determined as $x_{i - 1} + 4\left( {G - g} \right) = x_{i - 1} + 32$.
2. $x_{i}$ was increased by a given step size when $x_{i - 1_{p}} G$ was chosen over $x_{i_{p}} g$, and it was similarly decreased when $x_{i_{p}} g$ was chosen over $x_{i - 1_{p}} G$, as long as $x_{i} > x_{i - 1}$. In case of $x_{i} \le x_{i - 1}, \, x_{i}$ was increased to ensure outcome monotonicity.
3. The initial step was $4\left( {G - g} \right) = 32$, and the step sizes were halved after each choice.
4. The indifference point was reached after five choices.
5. The largest possible value of $x_{i}$ was $x_{i - 1} + 32 + 32 + 16 + 8 + 4 + 2 = x_{i - 1} + 94$.
6. The smallest possible value of $x_{i}$ was $x_{i - 1} + 32 - 32 + 16 - 8 - 4 - 2 = x_{i - 1} + 2$. The fourth term on the left-hand side (+16) ensured the monotonicity of outcomes (see point 2).

One concern about the TO method and the bisection iteration process is the method’s incentive compatibility, due to the adaptive design involved. A subject who is fully aware of the adaptive design can strategically drive the value $x_{i}$ upwards by pretending to be extremely risk-averse in response to the bisection questions. In this way, he or she can increase the expected values of prospects in the subsequent questions for the elicitation of $x_{i + 1}$. To make it more difficult for our subjects to grasp this process fully, we included two filler questions in the iteration process of each $x_{i}$. These two filler choices were placed after the first and the third choice questions for every $x_{i}$. In these questions, $x_{i}$ was changed in a direction opposite to that assumed in the changes described in point 2 above. These questions had no further impact on the flow of the procedure.

The filler questions permitted a further test of consistency, as they required preferences that were in line with the previous choices. This kind of preference was required because the preferred option in the previous choice question was made even more attractive in the filler questions. Consistency rates were as high as 97.5% in the first filler question, and 93.3% in the second filler question. The slight decrease of consistency in responses to the second question can possibly be explained by its being closer to the indifference point.

Our data did not suggest any strategic behavior. Although an awareness of the adaptive design from the outset was unlikely, it could be expected that learning during the experiment would lead to increasing distances between $x_{i}$ s. For example, this could lead to larger distances between $x_{5}$ and $x_{4}$ than between $x_{1}$ and $x_{0}$. However, the medians of these distances in our data were 26 and 34, respectively, and they did not differ significantly (Wilcoxon sign-rank, *p* value = 0.54).

## Electronic supplementary material

Below is the link to the electronic supplementary material.

Supplementary material 1 (PDF 2980 kb)

## Footnotes

1 A complete list of the evidence for and against the inverse S in the DFD literature is provided in Online Appendix 2.

2 By *sampling error*, we refer to a statistical error that arises when the observed sampling information is unrepresentative of the outcome distribution, but not to other types of errors that arises while processing the information. See Sect. 6.2 for further discussion.

3 The term “risk” designates situations where the knowledge of objective probabilities is available or in principle, attainable to the decision-makers as in the CSP. However, we acknowledge that uncertainty about probabilities may still arise in subjects’ judgments about the experienced probabilities based on their counting of the frequencies of outcomes. See Sect. 6.2 for further discussion.

4 We used the elicited $x_{1}$ as the minimum outcome of the risky prospects to avoid problems related to the extreme behavior of power utility near its origin (Wakker 2008), i.e., $x_{0}$ in our design. In particular, for $\alpha < 1$, the slope of the power utility converges to infinity as *x* tends to the origin. This implies extreme risk aversion near the origin. Similarly, $\alpha > 1$ implies extreme risk-seeking near the origin.

5 In our experiment, one subject reached the lowest possible bound of $x_{i}$’s in all five cases. This subject got ($x_{5} - x_{1} = 8$). Therefore, the resulting estimations, $s_{0.05} = x_{1}$ and $s_{0.95} = x_{5}$, made the preference for $x_{5_{0.05}} x_{1}$ over $s_{0.05}$ and the preference for $s_{0.95}$ over $x_{5_{0.95}} x_{1}$ trivial, due to the domination of the safe or the risky prospect. Consequently, her standard sequence was not spaced well enough to make the estimations of $s_{q}$ with Eq. (1). We excluded this subject from the following analysis. The analysis with this subject included does not alter our conclusions.

6 A post hoc analysis showed that the power function fits our data better than the exponential function $u\left( x \right) = 1 - e^{ - \theta x}$. The power function gave higher average $R^{2}$ values than the exponential function (mean values 0.985 vs. 0.959, $p < 0.01$, Wilcoxon test, two-sided).

7 The two treatment groups also did not differ in terms of age, gender, or nationality. The randomization checks are reported in Online Appendix 3.

8 The overweighting of good rare outcomes amounts to overweighting of 0.05, 0.10, and 0.20 whereas the overweighting of bad rare outcomes amounts to underweighting of 0.80, 0.90, and 0.95.

9 Bayes factors were computed with the package BayesFactor in R (Morey et al. 2015).

10 The same results were obtained with the compound invariance family of Prelec (1998). See our Online Appendix 6.

11 The notes are available at https://www.dropbox.com/s/yxttzvr6tdl8jr1/notes.pdf?dl=0.

12 Another factor that was discussed in the previous studies was the effect of recency. This factor was commonly formulated as the second half of the sequence predicting the choice behavior better than the first half. Our study did not detect any recency effect. See Online Appendix 9.

## References

[CR1] Abdellaoui M (2000). Parameter-free elicitation of utility and probability weighting functions. Management Science.

[CR2] Abdellaoui M, Baillon A, Placido L, Wakker PP (2011). The rich domain of uncertainty: Source functions and their experimental implementation. American Economic Review.

[CR3] Abdellaoui M, Bleichrodt H, Paraschiv C (2007). Loss aversion under prospect theory: A parameter-free measurement. Management Science.

[CR4] Abdellaoui M, L’Haridon O, Paraschiv C (2011). Experienced versus described uncertainty: Do we need two prospect theory specifications?. Management Science.

[CR5] Abdellaoui M, Vossmann F, Weber M (2005). Choice-based elicitation and decomposition of decision weights for gains and losses under uncertainty. Management Science.

[CR6] Allais M (1953). Le Comportement de l’Homme Rationnel devant le Risque: Critique des Postulats et Axiomes de l’Ecole Americaine. Econometrica.

[CR7] Ashby NJ, Rakow T (2014). Forgetting the past: Individual differences in recency in subjective valuations from experience. Journal of Experimental Psychology. Learning, Memory, and Cognition.

[CR8] Baillon A, Bleichrodt H, Liu N, Wakker PP (2016). Group decision rules and group rationality under risk. Journal of Risk and Uncertainty.

[CR9] Balcombe K, Fraser I (2015). Parametric preference functionals under risk in the gain domain: A Bayesian analysis. Journal of Risk and Uncertainty.

[CR10] Bardsley N, Cubitt R, Loomes G, Moffat P, Starmer C, Sugden R (2010). Experimental economics: Rethinking the rules.

[CR11] Barron G, Erev I (2003). Small feedback-based decisions and their limited correspondence to description-based decisions. Journal of Behavioral Decision Making.

[CR12] Barron G, Ursino G (2013). Underweighting rare events in experience based decisions: Beyond sample error. Journal of Economic Psychology.

[CR13] Binmore K (1999). Why experiment in economics?. The Economic Journal.

[CR14] Bleichrodt H, Cillo A, Diecidue E (2010). A quantitative measurement of regret theory. Management Science.

[CR15] Bleichrodt H, Filko M, Kothiyal A, Wakker PP (2017). Making case-based decision theory directly observable. American Economic Journal: Microeconomics.

[CR16] Bleichrodt H, Pinto JL (2000). A parameter-free elicitation of the probability weighting function in medical decision analysis. Management Science.

[CR17] Brandstätter E, Gigerenzer G, Hertwig R (2006). The priority heuristic: Making choices without trade-offs. Psychological Review.

[CR18] Broomell SB, Bhatia S (2014). Parameter recovery for decision modeling using choice data. Decision.

[CR19] Camerer CF, Ho T-H (1994). Violations of the betweenness axiom and nonlinearity in probability. Journal of Risk and Uncertainty.

[CR20] Camilleri AR, Newell BR (2009). The role of representation in experience-based choice. Judgment and Decision Making.

[CR21] Charness G, Karni E, Levin D (2007). Individual and group decision making under risk: An experimental study of Bayesian updating and violations of first-order stochastic dominance. Journal of Risk and Uncertainty.

[CR22] Cubitt, R., Kopsacheilis, O., & Starmer, C. (2019) An inquiry into the nature and causes of the Description—Experience gap, Centre for Decision Research and Experimental Economics, University of Nottingham, Working Paper 2019-15

[CR23] Dimmock SG, Kouwenberg R, Wakker PP (2016). Ambiguity attitudes in a large representative sample. Management Science.

[CR24] Ellsberg D (1961). Risk, ambiguity, and the savage axioms. The Quarterly Journal of Economics.

[CR25] Erev I, Ert E, Plonsky O, Cohen D, Cohen O (2017). From anomalies to forecasts: Toward a descriptive model of decisions under risk, under ambiguity, and from experience. Psychological Review.

[CR26] Ert E, Haruvy E (2017). Revisiting risk aversion: Can risk preferences change with experience?. Economics Letters.

[CR27] Ert E, Trautmann ST (2014). Sampling experience reverses preferences for ambiguity. Journal of Risk and Uncertainty.

[CR28] Etchart-Vincent N (2004). Is probability weighting sensitive to the magnitude of consequences? An experimental investigation on losses. Journal of Risk and Uncertainty.

[CR29] Etchart-Vincent N (2009). Probability weighting and the ‘level’ and ‘spacing’ of outcomes: An experimental study over losses. Journal of Risk and Uncertainty.

[CR30] Fehr-Duda H, Epper T (2012). Probability and risk: Foundations and economic implications of probability weighting. Annual Review of Economics.

[CR31] Fox CR, Hadar L (2006). “Decisions from experience” = sampling error + prospect theory: Reconsidering Hertwig, Barron, Weber & Erev (2004). Judgment and Decision Making.

[CR32] Fox CR, Tversky A (1998). A belief-based account of decision under uncertainty. Management Science.

[CR33] Gilboa I, Schmeidler D (1995). Case-based decision theory. The Quarterly Journal of Economics.

[CR34] Gilboa I, Schmeidler D (2001). A theory of case-based decisions.

[CR35] Glöckner A, Hilbig BE, Henninger F, Fiedler S (2016). The reversed description-experience gap: Disentangling sources of presentation format effects in risky choice. Journal of Experimental Psychology: General.

[CR36] Golan H, Ert E (2015). Pricing decisions from experience: The roles of information-acquisition and response modes. Cognition.

[CR37] Goldstein WM, Einhorn HJ (1987). Expression theory and the preference reversal phenomena. Psychological Review.

[CR38] Gonzalez R, Wu G (1999). On the shape of the probability weighting function. Cognitive Psychology.

[CR39] Grosskopf B, Sarin R, Watson E (2015). An experiment on case-based decision making. Theory and Decision.

[CR40] Gul F (1991). A theory of disappointment aversion. Econometrica.

[CR41] Hadar L, Fox CR (2009). Information asymmetry in decision from description versus decision from experience. Judgment and Decision Making.

[CR42] Hau R, Pleskac TJ, Hertwig R (2010). Decisions from experience and statistical probabilities: Why they trigger different choices than a priori probabilities. Journal of Behavioral Decision Making.

[CR43] Hau R, Pleskac TJ, Kiefer J, Hertwig R (2008). The description–experience gap in risky choice: The role of sample size and experienced probabilities. Journal of Behavioral Decision Making.

[CR44] Hertwig R (2012). The psychology and rationality of decisions from experience. Synthese.

[CR45] Hertwig R, Barron G, Weber EU, Erev I (2004). Decisions from experience and the effect of rare events in risky choice. Psychological Science.

[CR46] Hertwig, R., Barron, G., Weber, E. U., & Erev, I. (2006). The role of information sampling in risky choice. *Information Sampling and Adaptive Cognition*, 72–91.

[CR47] Hertwig R, Erev I (2009). The description–experience gap in risky choice. Trends in Cognitive Sciences.

[CR48] Hertwig R, Pleskac TJ (2010). Decisions from experience: Why small samples?. Cognition.

[CR49] Hills TT, Hertwig R (2010). Information search in decisions from experience: Do our patterns of sampling foreshadow our decisions?. Psychological Science.

[CR50] Holt CA, Laury SK (2002). Risk aversion and incentive effects. American Economic Review.

[CR51] Humphrey SJ (2006). Does learning diminish violations of independence, coalescing and monotonicity?. Theory and Decision.

[CR52] Jeffreys H (1961). The theory of probability.

[CR53] Kahneman, D., & Tversky, A. (1979). Prospect theory: An analysis of decisions under risk. *Econometrica*, 263–291.

[CR54] Kellen D, Pachur T, Hertwig R (2016). How (in)variant are subjective representations of described and experienced risk and rewards?. Cognition.

[CR55] Kemel E, Travers M (2016). Comparing attitudes toward time and toward money in experience-based decisions. Theory and Decision.

[CR56] Kopsacheilis O (2017). The role of information search and its influence on risk preferences. Theory and Decision.

[CR57] Kruschke, J. (2011). *Doing Bayesian data analysis: A tutorial with R, JAGS, and Stan*. Academic Press.

[CR58] Lejarraga T, Hertwig R, Gonzalez C (2012). How choice ecology influences search in decisions from experience. Cognition.

[CR59] Lejarraga T, Pachur T, Frey R, Hertwig R (2016). Decisions from experience: From monetary to medical gambles. Journal of Behavioral Decision Making.

[CR60] Loomes G, Starmer C, Sugden R (2003). Do anomalies disappear in repeated markets?. The Economic Journal.

[CR61] Morey, R. D., Rouder, J. N., & Jamil, T. (2015). BayesFactor: Computation of Bayes factors for common designs. *R package version 0.9, 9*, 2014.

[CR62] Nilsson H, Rieskamp J, Wagenmakers E-J (2011). Hierarchical Bayesian parameter estimation for cumulative prospect theory. Journal of Mathematical Psychology.

[CR63] Ossadnik W, Wilmsmann D, Niemann B (2013). Experimental evidence on case-based decision theory. Theory and Decision.

[CR64] Pachur T, Suter RS, Hertwig R (2017). How the twain can meet: Prospect theory and models of heuristics in risky choice. Cognitive Psychology.

[CR65] Palma AD, Abdellaoui M, Attanasi G, Ben-Akiva M, Erev I, Fehr-Duda H (2014). Beware of black swans: Taking stock of the description–experience gap in decision under uncertainty. Marketing Letters.

[CR66] Plott CR, Arrow KJ, Colombatto E, Perlaman M, Schmidt C (1996). Rational individual behavior in markets and social choice processes: The discovered preference hypothesis. The rational foundations of economic behaviour.

[CR67] Prelec D (1998). The probability weighting function. Econometrica.

[CR68] Qiu J, Steiger E-M (2010). Understanding the two components of risk attitudes: An experimental analysis. Management Science.

[CR69] Regenwetter M, Robinson MM (2017). The construct–behavior gap in behavioral decision research: A challenge beyond replicability. Psychological Review.

[CR70] Scheibehenne B, Pachur T (2015). Using Bayesian hierarchical parameter estimation to assess the generalizability of cognitive models of choice. Psychonomic Bulletin & Review.

[CR71] Schunk D, Betsch C (2006). Explaining heterogeneity in utility functions by individual differences in decision modes. Journal of Economic Psychology.

[CR72] Stott HP (2006). Cumulative prospect theory’s functional menagerie. Journal of Risk and Uncertainty.

[CR73] Taleb NN (2007). The black swan: The impact of the highly improbable.

[CR74] Thaler RH, Sunstein CR (2008). Nudge: Improving decisions about health, wealth, and happiness.

[CR75] Trautmann ST, Van De Kuilen G (2015). Ambiguity attitudes. The Wiley Blackwell handbook of Judgment and Decision Making.

[CR76] Tversky A, Fox CR (1995). Weighing risk and uncertainty. Psychological Review.

[CR77] Tversky A, Kahneman D (1981). The framing of decisions and the psychology of choice. Science.

[CR78] Tversky A, Kahneman D (1992). Advances in prospect theory: Cumulative representation of uncertainty. Journal of Risk and Uncertainty.

[CR79] Ungemach C, Chater N, Stewart N (2009). Are probabilities overweighted or underweighted when rare outcomes are experienced (Rarely)?. Psychological Science.

[CR80] van de Kuilen G (2009). Subjective probability weighting and the discovered preference hypothesis. Theory and Decision.

[CR81] van de Kuilen G, Wakker PP (2006). Learning in the Allais paradox. Journal of Risk and Uncertainty.

[CR82] van de Kuilen G, Wakker PP (2011). The midweight method to measure attitudes toward risk and ambiguity. Management Science.

[CR83] von Neumann J, Morgenstern O (1944). The theory of games and economic behavior.

[CR84] Wakker PP (2004). On the composition of risk preference and belief. Psychological Review.

[CR85] Wakker PP (2008). Explaining the characteristics of the power (CRRA) utility family. Health Economics.

[CR86] Wakker PP (2010). Prospect theory: For risk and ambiguity.

[CR87] Wakker PP, Deneffe D (1996). Eliciting von Neumann–Morgenstern utilities when probabilities are distorted or unknown. Management Science.

[CR88] Wakker PP, Erev I, Weber EU (1994). Comonotonic independence: The critical test between classical and rank-dependent utility theories. Journal of Risk and Uncertainty.

[CR89] Weber EU, Shafir S, Blais A-R (2004). Predicting risk sensitivity in humans and lower animals: Risk as variance or coefficient of variation. Psychological Review.

[CR90] Wulff DU, Mergenthaler Canseco M, Hertwig R (2018). A meta-analytic review of two modes of learning and the description-experience gap. Psychological Bulletin.

[CR91] Yechiam E, Busemeyer JR (2006). The effect of foregone payoffs on underweighting small probability events. Journal of Behavioral Decision Making.

[CR92] Zeisberger S, Vrecko D, Langer T (2012). Measuring the time stability of Prospect Theory preferences. Theory and Decision.

