# Supplementary material for: Experience and rationality under risk: re-examining the impact of sampling experience
Source: Exp Econ. 2020 Jan 16;23(4):1100–28. doi: 10.1007/s10683-019-09641-y (PMC7655602; doi:10.1007/s10683-019-09641-y)
Supplement: Supplementary file 1 — Supplementary material 1 (PDF 2980 kb) [file 10683_2019_9641_MOESM1_ESM.pdf]

Online Appendices of

Experience and Rationality under Risk: A Re-examination of the Description-Experience Gap

Ilke Aydogan[1], Yu Gao[2]

Online Appendix 1: Experimental Instructions

Instructions : Part 1

In this part of the experiment, you will face a series of choice questions involving choices between two prospects. Examples of choices are presented below. Risk is generated by throwing two ten-faced dice and adding the results together. One dice has the values 00, 10, …, 90 and the other has the values 0, 1, ..., 9. Thus the sum yields a random number between 0 up to 99, and each of these numbers is equally likely. For instance, in Figure 1 the random number generated by the two dice is 30+8=38.

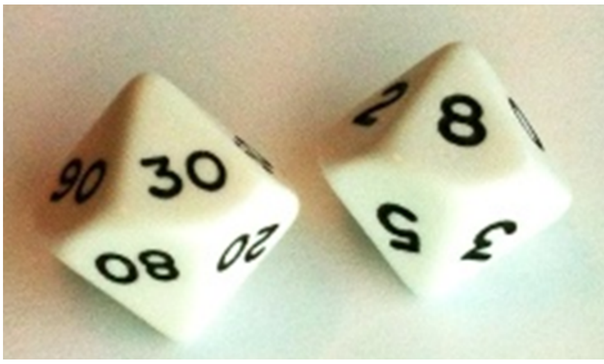

Figure 1

Example
Figure 2 presents a choice situation you will face in the experiment.

[1] IESEG School of Management, Lille, France; Department of Economics, Bocconi University, Milan, Italy. i.aydogan@ieseg.fr; aydogan@unibocconi.it

[2] Department of Applied Economics, Guanghua School of Management (GSM), Peking University, Beijing, 100871, China. ygao@gsm.pku.edu.cn

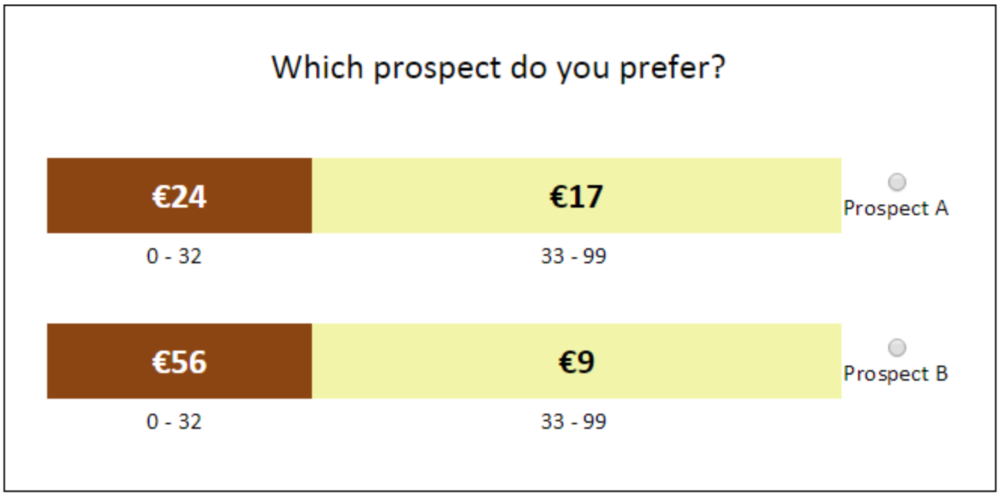

Figure 2

In this situation, you will be asked to choose between two prospects. Prospect A pays €24 if the dice give a number between 0 and 32 and €17 if the dice give a number between 33 and 99. Prospect B pays €56 if the dice give a number between 0 and 32 and €9 if the dice give a number between 33 and 99. You can choose your preferred prospect by clicking on the button next to it.

After you made your choice, a 'Confirm' button will appear. Please click on it to proceed. Before you press the "Confirm" button, you may change your choice between Prospect A and B. But once you press the "Confirm" button, you can no longer go back to change your choice.

We will now test your understanding of the instructions.

Assume you have been selected as one of the two participants who can play a question for real and that the question below was randomly selected.

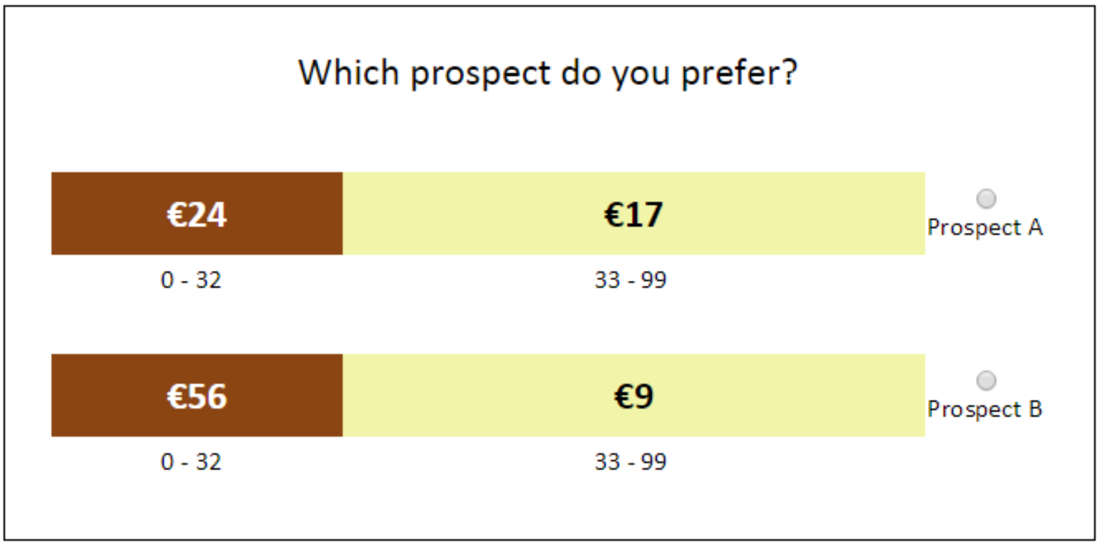

Figure 3

Please answer the following questions.

Question 1

If you chose prospect A, and the number generated by the dice is 61, then how much will you receive from this prospect?

- €24
- €56
- €9
- €17

Question 2

If you chose prospect B, and the number generated by the dice is 61, then how much will you receive from this prospect?

- €24
- €56
- €9
- €17

After answering the above comprehensive questions correctly, subjects will be directed to part 2.

In part 2 of the experiment, subjects will be randomly assigned to either the DFD treatment or the DFE treatment.

The following is the instructions for the DFD treatment.

Instructions: Part 2

In this part of the experiment, you will face 28 choice questions, involving choices between two prospects. Risk is generated by a random draw, **without looking**, from an urn containing balls with monetary values written on them. Figure 1 shows the urns that we are going to use.

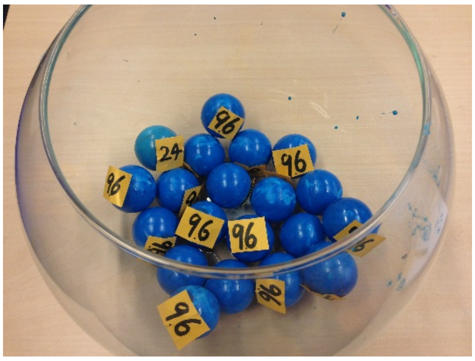

Figure 1

In each question, you will face two urns that contain balls with different values. You are asked to select the urn from which you prefer to draw a single ball randomly. The value of the ball drawn from the selected urn will determine your payment.

Example

Figure 2 presents a choice situation that you will face during the experiment. The left and right illustrations represent the urns containing balls. The total number of balls in each urn is shown. In this case each urn contains 20 balls. All other relevant information about the content of the urns is presented below the

illustrations. In this case, the left urn contains 19 balls each with value of €96 and 1 ball with value of €24 whereas the right urn contains 20 balls each with value of €92.

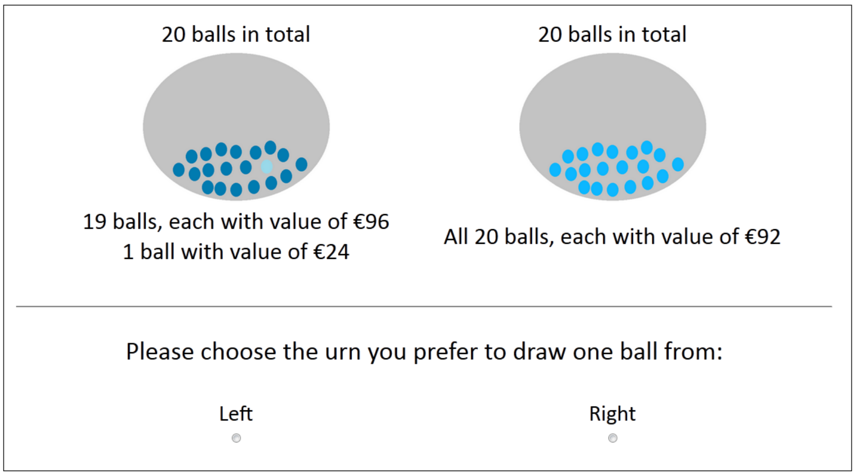

Figure 2

You will choose the urn that you prefer to draw a ball from by selecting "Left" or "Right". After you made your choice, a "Confirm" button will appear. Please click on it to proceed to the next question. Before you press the "Confirm" button, you can change your choice as many times as you like. But once you press the "Confirm" button, you can no longer go back to change your choice.

At the end of the experiment, if you are selected to play a choice question in this part for real, the urn that you selected in that question will be prepared by the experimenter in front of you. The urn will be then covered, and you will draw one ball from the urn without looking inside. The single ball that you draw from the urn will determine your payment.

Next

The following is the instructions for the DFE treatment.

Instructions: Part 2

In this part of the experiment, you will face 7 choice questions, involving choices between two prospects. Risk is generated by a random draw, **without looking**, from an urn containing balls with monetary values written on them. Figure 1 shows the urns that we are going to use.

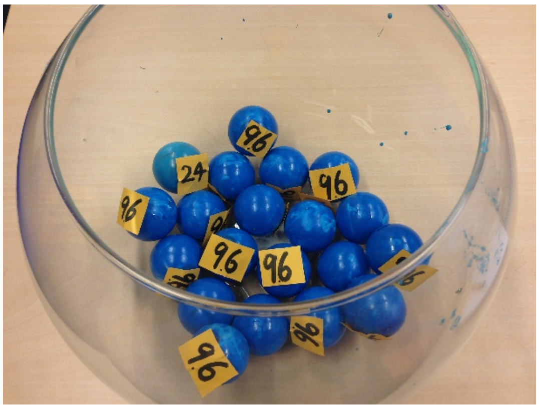

Figure 1

In each question, you will face two urns that contain balls with different values. You are asked to select the urn from which you prefer to draw a single ball randomly. The value of the ball drawn from the selected urn will determine your payment.

Example

Figure 2 presents a choice situation you will face during the experiment. The left and right illustrations represent the urns containing balls. The total number of balls in each urn is shown. In this case each urn contains 20 balls.

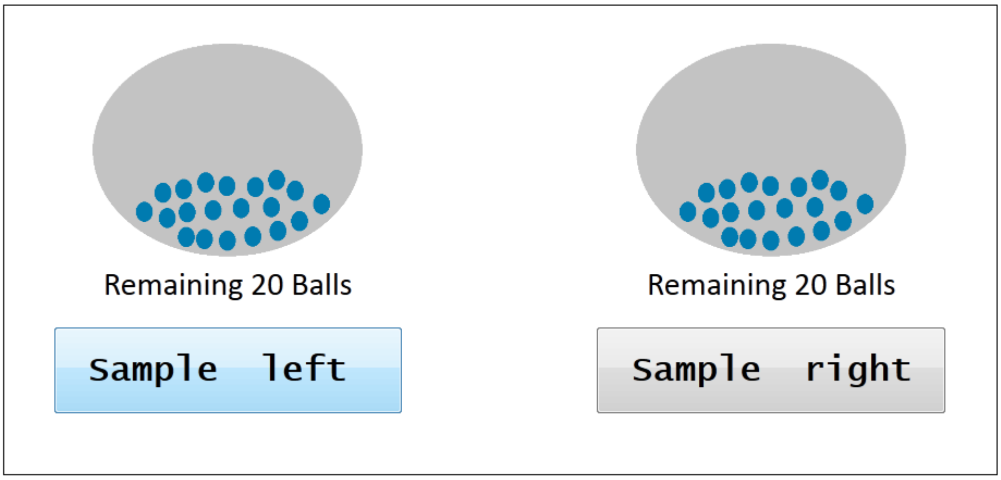

Figure 2

Before you choose the urn that you prefer to draw a ball from, you will learn about the values of balls in both urns. For this, you are asked to sample balls one by one from each urn by clicking the corresponding button "sample left" or "sample right", shown in Figure 2. The value of each ball sampled will be presented on the screen for 2 seconds immediately after each click. For example, if you click on the "sample right" button, you will see the screen in Figure 3 which tells you the monetary value of the ball draw is €27.

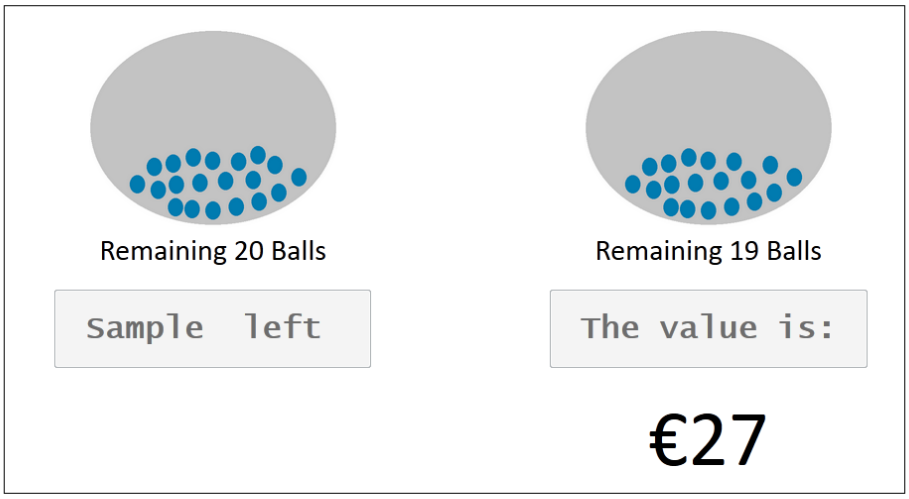

Figure 3

The sampling will be without replacement. It means that a ball drawn from the urn will not be put back into the urn. The number of balls remaining in urns will be updated on the screen after each draw. You can sample in whichever order you like. In case you want to take notes of the values that you observe, paper and pen are provided on your desk.

When both urns are empty, and you have observed the value of every ball in both urns, a "Proceed to the choice stage" button will be shown. By clicking on it, the balls will be returned into the urn where they belong. Then you will be directed to the choice stage. The resulting screen is shown in Figure 4.

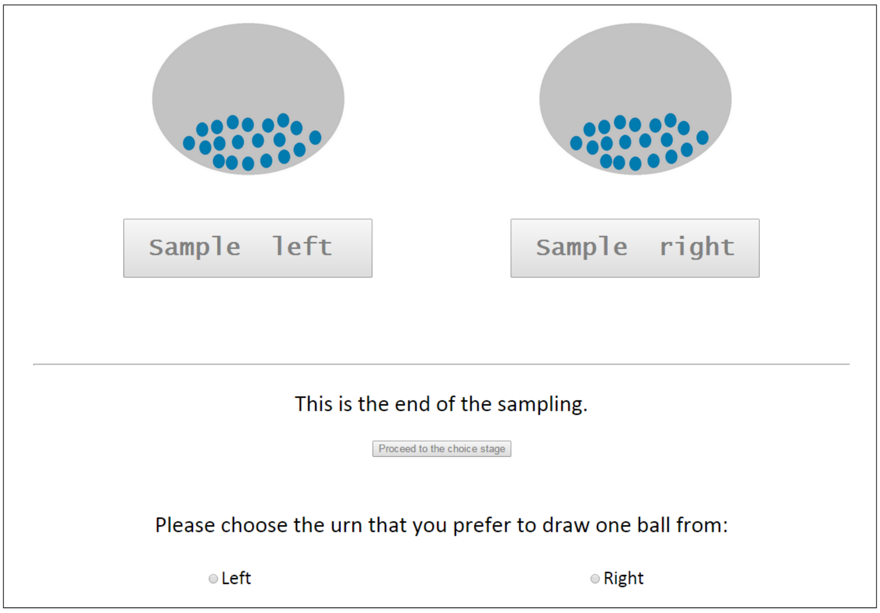

Figure 4

After you made your choice, a "Confirm" button will appear. Please click on it to proceed to the next question. Before you press the "Confirm" button, you can change your choice as many times as you like. But once you press the "Confirm" button, you can no longer go back to change your choice.

At the end of the experiment, if you are selected to play a choice question in this part for real, the urn that you selected in that question will be prepared by the experimenter in front of you. Then the single ball that you will draw from the urn will determine your payment.

By clicking "Next", you will be directed to a sample question which will familiarize you with the experimental questions.

Next

Online Appendix 2: Evidence for and against Inverse-S Probability Weighting for Risk

17 studies finding evidence against inverse-S

Alarie, Yves & Georges Dionne (2001) "Lottery Decisions and Probability Weighting Function," *Journal of Risk and Uncertainty 22,* 21–33.

Armantier, Olivier & Nicolas Treich (2009) "Subjective Probabilities in Games: An Application to the Overbidding Puzzle," *International Economic Review 50,* 1013–1041.

Balcombe, Kelvin & Iain Fraser (2015) "Parametric Preference Functionals under Risk in the Gain Domain: A Bayesian Analysis," *Journal of Risk and Uncertainty 50*, 161–187.

Birnbaum, Michael H. (2004) "Causes of Allais Common Consequence Paradoxes: An Experimental Dissection," *Journal of Mathematical Psychology 48*, 87–106.

Birnbaum, Michael H. & Darin Beeghley (1997) "Violations of Branch Independence in Judgments of the Value of Gambles," *Psychological Science 8,* 87–94.

Birnbaum, Michael H. & Alfredo Chavez (1997) "Tests of Theories of Decision Making: Violations of Branch Independence and Distribution Independence," *Organizational Behavior and Human Decision Processes 71*, 161–194.

Birnbaum, Michael H. & William R. McIntosh (1996) "Violations of Branch Independence in Choices between Gambles," *Organizational Behavior and Human Decision Processes 67,* 91–110.

Birnbaum, Michael H. & Juan B. Navarrete (1998) "Testing Descriptive Utility Theories: Violations of Stochastic Dominance and Cumulative Independence," *Journal of Risk and Uncertainty 17*, 49–78.

Goeree, Jacob K., Charles A. Holt, & Thomas R. Palfrey (2002) "Quantal Response Equilibrium and Overbidding in Private-Value Auctions," *Journal of Economic Theory 104,* 247–272.

Henrich, Joseph & Richard Mcelreat (2002) "Are Peasants Risk-Averse Decision Makers?" *Current Anthropology 43*, 172–181.

Humphrey, Steven J. & Arjan Verschoor (2004) "The Probability Weighting Function: Experimental Evidence from Uganda, India and Ethiopia," *Economics Letters 84,* 419–425.

Humphrey, Steven J. & Arjan Verschoor (2004) "Decision-Making under Risk among Small Farmers in East Uganda," *Journal of African Economies 13,* 44–101.

Jullien, Bruno & Bernard Salanié (2000) "Estimating Preferences under Risk: The Case of Racetrack Bettors," *Journal of Political Economy 108,* 503–530.

Krawczyk, M. W. (2015). Probability weighting in different domains: The role of affect, fungibility, and stakes. *Journal of Economic Psychology, 51,* 1-15.

Loomes, Graham (1991) "Evidence of a New Violation of the Independence Axiom," *Journal of Risk and Uncertainty 4,* 92–109.

Qiu, Jianying & Eva-Maria Steiger (2011) "Understanding the Two Components of Risk Attitudes: An Experimental Analysis," *Management Science 57,* 193–199.

van de Kuilen, Gijs & Peter P. Wakker (2011) "The Midweight Method to Measure Attitudes toward Risk and Ambiguity," *Management Science 57,* 582–598.

68 studies finding evidence supporting inverse-S

Abdellaoui, Mohammed (2000) "Parameter-Free Elicitation of Utility and Probability Weighting Functions," *Management Science* 46, 1497–1512.

Abdellaoui, Mohammed, Aurélien Baillon, Laetitia Placido, & Peter P. Wakker (2011) "The Rich Domain of Uncertainty: Source Functions and Their Experimental Implementation," *American Economic Review 101,* 695–723.

Abdellaoui, Mohammed, Han Bleichrodt, & Olivier L'Haridon (2008) "A Tractable Method to Measure Utility and Loss Aversion under Prospect Theory," *Journal of Risk and Uncertainty 36,* 245–266.

Abdellaoui, Mohammed, Olivier L'Haridon, & Corina Paraschiv (2011) "Experienced versus Described Uncertainty: Do We Need Two Prospect Theory Specifications?," *Management Science 57,* 1879–1895.

Abdellaoui, Mohammed & Emmanuel Kemel (2014) "Eliciting Prospect Theory when Consequences Are Measured in Time Units: "Time Is not Money," Management Science, forthcoming.

Ali, Mukhtar M. (1977) "Probability and Utility Estimates for Racetrack Betting," *Journal of Political Economy* 85, 803–815.

Allais, Maurice (1988) "The General Theory of Random Choices in Relation to the Invariant Cardinal Utility Function and the Specific Probability Function." *In* Bertrand R. Munier (ed.) *Risk, Decision and Rationality*, 233–289, Reidel, Dordrecht.

Barseghyan, Levon, Francesca Molinari, Ted O'Donoghue, & Joshua C. Teitelbaum (2013) "The Nature of Risk Preferences: Evidence from Insurance Choices," *American Economic Review 103*, 2499–2529.

Beach, Lee R. & Lawrence D. Phillips (1967) "Subjective Probabilities Inferred from Estimates and Bets," *Journal of Experimental Psychology* 75, 354–359.

Bernasconi, Michele (1994) "Nonlinear Preference and Two-stage Lotteries: Theories and Evidence," *Economic Journal* 104, 54–70.

Berns, Gregory S., C. Monica Capra, Jonathan Chappelow, Sara Moore, & Charles Noussair (2008) "Neurobiological Regret and Rejoice Functions for Aversive Outcomes," *Neuroimage* 39, 2047–2057.

Bleichrodt, Han & José Luis Pinto (2000) "A Parameter-Free Elicitation of the Probability Weighting Function in Medical Decision Analysis," *Management Science* 46, 1485–1496.

Booij, Adam S., Bernard M.S. Van Praag, & Gijs van de Kuilen (2010) "A Parametric Analysis of Prospect Theory's Functionals," *Theory and Decision 68,* 115–148.

Botzen, Wouter W. J., Howard Kunreuther, & Erwann Michel-Kerjan (2015) "Divergence between Individual Perceptions and Objective Indicators of Tail Risks: Evidence from Floodplain Residents in New York City," *Judgment and Decision Making 10,* 365–385.

Brandstätter, Eduard, Anton Kühberger, & Friedrich Schneider (2002) "A Cognitive-Emotional Account of the Shape of the Probability Weighting Function," *Journal of Behavioral Decision Making 15,* 79–100.

Bruhin, Adrian, Helga Fehr-Duda, & Thomas Epper (2010) "Risk and Rationality: Uncovering Heterogeneity in Probability Distortion," *Econometrica 78,* 1375–1412.

Budescu, David V., Han-Hui Por, Stephen B. Broomell, & Michael Smithson (2014) "The Interpretation of IPCC Probabilistic Statements around the World," *Nature Climate Change 4,* 508–512.

Camerer, Colin F. & Teck-Hua Ho (1994) "Violations of the Betweenness Axiom and Nonlinearity in Probability," *Journal of Risk and Uncertainty* 8, 167–196.

Chipman, John S. (1960) "Stochastic Choice and Subjective Probability." *In* Dorothy Willner (ed.) *Decisions, Values and Groups Vol. 1*, 70–95, Pergamon Press, New York.

Cicchetti, Charless J. & Jeffrey A. Dubin (1994) "A Microeconometric Analysis of Risk Aversion and the Decision to Self-Insure," *Journal of Political Economy* 102, 169–186.

Cohen, Michèle & Jean-Yves Jaffray (1988) "Certainty Effect versus Probability Distortion: An Experimental Analysis of Decision Making under Risk," *Journal of Experimental Psychology: Human Perception and Performance* 14, 554–560.

Cohen, Michèle, Jean-Yves Jaffray, & Tanios Said (1987) "Experimental Comparisons of Individual Behavior under Risk and under Uncertainty for Gains and for Losses," *Organizational Behavior and Human Decision Processes* 39, 1–22.

Donkers, A.C.D., Bertrand Melenberg, & Arthur H.O. van Soest (2001) "Estimating Risk Attitudes Using Lotteries; A Large Sample Approach," *Journal of Risk and Uncertainty* 22, 165–195.

Epper, Thomas, Helga Fehr-Duda, & Adrian Bruhin (2011) "Viewing the Future through a Warped Lens: Why Uncertainty Generates Hyperbolic Discounting," *Journal of Risk and Uncertainty* 43, 163–203.

Etchart, Nathalie (2004) "Is Probability Weighting Sensitive to the Magnitude of Consequences? An Experimental Investigation on Losses," *Journal of Risk and Uncertainty* 28, 217–235.

Etchart, Nathalie (2009) "Probability Weighting and the 'Level' and 'Spacing' of Outcomes: An Experimental Study over Losses," *Journal of Risk and Uncertainty 39,* 45–63.

Etchart, Nathalie & Olivier l'Haridon (2011) "Monetary Incentives in the Loss Domain and Behavior toward Risk: An Experimental Comparison of Three Reward Schemes Including Real Losses," *Journal of Risk and Uncertainty 42,* 61–83.

Fehr-Duda, Helga, Adrian Bruhin, Thomas Epper & Renate Schubert (2010) "Rationality on the Rise: Why Relative Risk Aversion Increases with Stake Size," *Journal of Risk and Uncertainty 40,* 147–180.

Fehr-Duda, Helga & Thomas Epper (2012) "Probability and Risk: Foundations and Economic Implications of Probability-Dependent Risk Preferences," *Annual Review of Economics 4,* 567–593.

Fehr-Duda, Helga, Thomas Epper, Adrian Bruhin, & Renate Schubert (2011) "Risk and Rationality: The Effects of Mood and Decision Rules on Probability Weighting," *Journal of Economic Behavior and Organization* 78, 14–24.

Fehr-Duda, Helga, Manuele de Gennaro, & Renate Schubert (2006) "Gender, Financial Risk, and Probability Weights," *Theory and Decision* 60, 283–313.

Gonzalez, Richard & George Wu (1999) "On the Shape of the Probability Weighting Function," *Cognitive Psychology* 38, 129–166.

Green, Leonard, & Joel Myerson (2004) "A Discounting Framework for Choice with Delayed and Probabilistic Rewards," *Psychological Bulletin* 130, 769-792.

Griffith, Richard M. (1949) "Odds Adjustments by American Horse Race Bettors," *American Journal of Psychology* 62, 290–294.

Griffith, Richard M. (1961) "A Footnote on Horse Race Betting," *Transactions Kentucky Academic Science* 22, 78–81.

Kachelmeier, Steven J. & Mohamed Shehata (1992) "Examining Risk Preferences under High Monetary Incentives: Experimental Evidence from the People's Republic of China," *American Economic Review* 82, 1120–1141; for comment see Steven J. Kachelmeier & Mohammed Shehata (1994) *American Economic Review* 84, 1104–1106.

Kahneman, Daniel & Amos Tversky (1979) "Prospect Theory: An Analysis of Decision under Risk," *Econometrica* 47, 263–291.

Kemel, Emmanuel & Corina Paraschiv (2014) "Prospect Theory for joint Time and Money Consequences in Risk and Ambiguity," *Transportation Research Part B: Methodological 50,* 81–95.

Kilka, Michael & Martin Weber (2001) "What Determines the Shape of the Probability Weighting Function under Uncertainty," *Management Science* 47, 1712–1726.

Krzysztofowicz, Roman (1994) "Generic Utility Theory: Explanatory Model, Behavioral Hypotheses, Empirical Evidence." *In* Maurice Allais & Ole Hagen (eds.) "C*ardinalism;* A Fundamental Approach," 249–288, Kluwer Academic Publishers, Dordrecht.

Kusev, Petko, Paul van Schaik, Peter Ayton, John Dent, & Nick Chater (2009) "Exaggerated Risk: Prospect Theory and Probability Weighting In Risky Choice," *Journal of Experimental Psychology: Learning, Memory, and Cognition 35,* 1487–1505.

Lattimore, Pamela M., Joanna R. Baker, & Ann D. Witte (1992) "The Influence of Probability on Risky Choice," *Journal of Economic Behavior and Organization* 17, 377–400.

Loehman, Edna (1998) "Testing Risk Aversion and Nonexpected Utility Theories," *Journal of Economic Behavior and Organization* 33, 285–302.

Luce, R. Duncan & Patrick Suppes (1965) "Preference, Utility, and Subjective Probability." *In* R. Duncan Luce, Robert R. Bush, & Eugene Galanter (eds.) *Handbook of Mathematical Psychology*, Vol. III, 249–410, Wiley, New York.

McGlothlin, William H. (1956) “Stability of Choices among Uncertain Alternatives,” *American Journal of Psychology* 69, 604–615.

Mukherjee, Kanchan (2011) “Thinking Styles and Risky Decision-Making: Further Exploration of the Affect–Probability Weighting Link,” *Journal of Behavioral Decision Making 24,* 443–455.

Petrova, Dafina G., Joop van der Pligt, & Rocio Garcia-Retamero (2014) “Feeling the Numbers: On the Interplay Between Risk, Affect, and Numeracy,” *Journal of Behavioral Decision Making 27,* 191–199.

Polkovnichenko, Valery & Feng Zhao (2013) “Probability Weighting Functions Implied in Options Prices,” *Journal of Financial Economics* 107, 580–609.

Preston, Malcolm G. & Philip Baratta (1948) “An Experimental Study of the Auction Value of an Uncertain Outcome,” *American Journal of Psychology* 61, 183–193.

Quiggin, John (1981) “Risk Perception and Risk Aversion among Australian Farmers,” *Australian Journal of Agricultural Economics* 25, 160–169.

Riddel, Mary (2012) “Comparing Risk Preferences over Financial and Environmental Lotteries,” *Journal of Risk and Uncertainty 45,* 135–157.

Riddel, Mary & Sonja Kolstoe (2013) “Heterogeneity in Life-duration Preferences: Are Risky Recreationists Really More Risk Loving,” *Journal of Risk and Uncertainty 46,* 191–213.

Rosett, Richard N. (1965) “Gambling and Rationality,” *Journal of Political Economy* 73, 595–607.

Rosett, Richard N. (1971) “Weak Experimental Verification of the Expected Utility Hypothesis,” *Review of Economic Studies* 38, 481–492.

Shaefer, Ralf E. & Katrin Borcherding (1973) “The Assessment of Subjective Probability Distributions: A Training Experiment,” *Acta Psychologica* 37, 117–129.

Sprowls, R. Clay (1953) “Psychological-Mathematical Probability in Relationships of Lottery Gambles,” *American Journal of Psychology* 66, 126–130.

Stalmeier, Peep F.M. & Thom G.G. Bezembinder (1999) “The Discrepancy between Risky and Riskless Utilities: A Matter of Framing?,” *Medical Decision Making*, 19, 435–447.

Tversky, Amos (1967) “Utility Theory and Additivity Analysis of Risky Choices,” *Journal of Experimental Psychology* 75, 27–36.

Tversky, Amos & Craig R. Fox (1995) “Weighing Risk and Uncertainty,” *Psychological Review* 102, 269–283.

Tversky, Amos & Daniel Kahneman (1992) “Advances in Prospect Theory: Cumulative Representation of Uncertainty,” *Journal of Risk and Uncertainty* 5, 297–323.

van de Stadt, Huib, Gerrit Antonides, & Bernard M.S. van Praag (1984) “Empirical Testing of the Expected Utility Model,” *Journal of Economic Psychology* 5, 17–29.

Viscusi, W. Kip & William N. Evans (2006) “Behavioral Probabilities,” *Journal of Risk and Uncertainty* 32, 5–15.

Winkler, Robert L. (1967) “The Assessment of Prior Distributions in Bayesian Analysis,” *Journal of the American Statistical Association* 62, 776–800.

Wu, George & Richard Gonzalez (1996) "Curvature of the Probability Weighting Function," *Management Science* 42, 1676–1690.

Wu, George & Richard Gonzalez (1998) "Common Consequence Conditions in Decision Making under Risk," *Journal of Risk and Uncertainty* 16, 115–139.

Wu, George & Richard Gonzalez (1999) "Nonlinear Decision Weights in Choice under Uncertainty," *Management Science* 45, 74–85.

Yaari, Menahem E. (1965) "Convexity in the Theory of Choice under Risk," *Quarterly Journal of Economics* 79, 278–290.

Zeisberger, Stefan, Dennis Vrecko, & Thomas Langer (2012) "Measuring the Time Stability of Prospect Theory Preferences," *Theory and Decision 72,* 359–386.

Online Appendix 3: Randomization check

	DFD	DFE	Kolmogorov-Smirnov tests p-values	Wilcoxon tests p-values
**X1**	62.09	58.56	0.99	0.54
**X2**	90.31	90.42	0.99	0.92
**X3**	123.24	127.30	0.73	0.83
**X4**	161.96	166.51	0.88	0.85
**X5**	202.18	206.19	0.71	0.91
**Utility curvature**	0.98	1.03	0.99	0.84
**male**	53.5%	55.6%	/	0.85
**age**	23	23	/	0.98
**Nation (Dutch or not)**	50%	53.5%	/	0.75

In the first two columns, reported are mean values

Online Appendix 4: Empirical Findings on Probability Weighting under DFE

Study	Sampling Experience	Sampling error & Information Asymmetry*	Analysis: Level & Type	Finding	Notes
**Hau *et al.* (2008)** Experiment 1	Autonomous sampling	Both present	Aggregate level – parametric	Approximately linear probability weighting	The analysis was based on a small set of choice problems. Parametric analysis was based on pooled data. Observed relative frequencies were used in the analysis.
Experiment 2	Autonomous sampling	Sampling error is reduced, information asymmetry is present	Aggregate level – parametric		
Experiment 3	Regulated sampling	Sampling error is reduced, Information asymmetry is present	Aggregate level – parametric		
**Ungemach *et al.* (2009)** Experiment 1	Autonomous sampling	Both present	Aggregate level – parametric	S-shaped probability weighting	The analysis was based on a small set of choice problems. Authors reported ranges of parameters providing the best fit to the data. Observed relative frequencies and judged probabilities (elicited in Experiment 2) were used in the analysis.
	Regulated sampling	Sampling error is eliminated, Information asymmetry is present	Aggregate level – parametric	Approximately linear probability weighting	
Experiment 2	Regulated sampling	Sampling error is eliminated, Information asymmetry is present	Aggregate level – parametric	S-shaped probability weighting	
**Abdellaoui *et al.* (2011)**	Autonomous sampling	Sampling error is present, Information asymmetry is reduced	Aggregate level – both nonparametric and parametric	Inverse-S shaped probability weighting, less pronounced under DFE than under DFD	Subjects were provided with descriptive information about the set of possible outcomes in prospects. Measures were based on certainty equivalent data. Observed relative frequencies were used in the analysis. The problem of aggregation was avoided by using linear interpolations of the weighting of probabilities of interest at the individual level.

Table Continued

Study	Sampling Experience	Sampling error & Information Asymmetry*	Analysis: Level & Type	Finding	Notes
**Frey *et al.* (2015)** Experiment 2	Autonomous sampling	Both present	Individual level - parametric	S-shaped probability weighting	Observed relative frequencies were used in the analysis.
**Kemel and Travers (2016)** Experiment 1	Autonomous sampling	Both present	Aggregate level – parametric	Inverse S-shaped probability weighting	Subjects were provided with descriptive information about the set of possible outcomes in Experiment 2. Measures were based on certainty equivalent data. The analysis was done both by using observed relative frequencies and unknown objective probabilities. Heterogeneity at the individual level was controlled by mixed modeling.
Experiment 2	Autonomous sampling	Sampling error is present, Information asymmetry is reduced	Aggregate level – parametric	Inverse S-shaped probability weighting	
**Glöckner *et al.* (2016)** Reanalysis of Glöckner et al. (2012)	Autonomous Sampling	Sampling error is present, Information asymmetry is reduced	Aggregate level – parametric	Inverse-S shaped probability weighting, more pronounced under DFE than under DFD	Subjects were provided with information about the number of possible outcomes in prospects, except for half of the subjects in Experiment 2. No difference in probability weighting was detected due to the absence of information in Experiment 2. Choice problems did not involve sure outcomes in the study of Glöckner et al. (2012) and in experiment 2. Only a minority of choice problems involved a sure outcome in experiments 1 and 3. Observed relative frequencies were used in the analysis.
Experiment 1	Autonomous Sampling	Sampling error is present, Information asymmetry is reduced	Aggregate level – parametric	Inverse-S shaped probability weighting, more pronounced under DFE than under DFD	
Experiment 2	Autonomous Sampling	Sampling error is present, Information asymmetry is reduced	Aggregate level – parametric	Inverse-S shaped probability weighting, more pronounced under DFE than under DFD	
Experiment 3	Autonomous Sampling	Sampling error is present, Information asymmetry is reduced	Aggregate & individual level – parametric	Inverse-S shaped probability weighting, more pronounced under DFE than under DFD	

Table Continued

Study	Sampling Experience	Sampling error & Information Asymmetry*	Analysis: Level & Type	Finding	Notes
Reanalysis of Erev *et al.* (2010)	Autonomous Sampling	Both present	Aggregate level – parametric	Approximately linear probability weighting	Every choice problem involved a two-outcome risky prospect and a sure outcome. Observed relative frequencies were used in the analysis.
**Kellen *et al.* (2016)**	Autonomous sampling	Both present	Aggregate & individual level – parametric	Inverse-S shaped probability weighting, more pronounced under DFE than under DFD	Observed relative frequencies were used in the analysis. Estimations were done with Bayesian Hierarchical modeling.
**Regenwetter and Robinson (2017)** Reanalysis of Hertwig *et al.* (2004), Hau *et al.* (2008) and Ungemach *et al.* (2009)	See Hertwig *et al.* (2004), Hau *et al.* (2008) and Ungemach *et al.* (2009)	See Hertwig *et al.* (2004), Hau *et al.* (2008) and Ungemach *et al.* (2009)	Aggregate level analysis – parametric	S-shaped probability weighting	The analysis was based on unknown objective probabilities rather than observed relative frequencies. Choice patterns were analyzed according to their compatibility with inverse S-shaped probability weighting. Individual heterogeneity was controlled in statistical analysis

** Here, sampling error describes deviations of observed relative frequencies from underlying objective probabilities, and information asymmetry describes the distinction between unknown vs. known objective probabilities in DFE and DFD respectively. In the studies documented in the table, information asymmetry remains between DFE and DFD, even though the sampling error is eliminated, as the sampling is done with replacement. Some studies reduce the information asymmetry by providing information about possible outcomes in prospects, which reveals certainty or possibility of outcomes.*

Online Appendix 5: Bayesian Hierarchical Estimation Procedure

We implemented Bayesian hierarchical estimation procedure as follows. The Goldstein and Einhorn (1987) probability weighting function is $w(q) = \frac{\delta q^{\gamma}}{\delta q^{\gamma}+(1-q)^{\gamma}}$. The probability of choosing the risky prospect was calculated using Luce (1959) stochastic choice function, which gave a better fit to our data than the logit function. It is $\Pr\,(choosing\ risky\ option) = \frac{RDU_{risky}^{\varphi}}{RDU_{risky}^{\varphi}+RDU_{safe}^{\varphi}}$, where φ is the noise parameter. After normalizing $U(x_1) = 0$, and $U(x_5) = 1$; $RDU_{risky} = w(q) * U(x_5) + \big(1 - w(q)\big) * U(x_1) = w(q)$, and $RDU_{safe} = U\big(x_q\big) = q$ by construction. Thus, the choice function implies random choice when $w(q) = q$, consistent with (3) in the Method section.

In the estimations, the individual level parameters γ_i and δ_i were constrained by using plausible ranges based on the previous findings in the literature and on the findings from our nonparametric analysis. Given the limitation of the dataset, and especially limited number of observations at the individual level, to ensure the identifiability, mildly small ranges were used for constraining the individual level parameters. The ranges of the prior distributions were from 0.1 to 2 for γ_i and from 0.1 to 1.5 for δ_i. The range chosen for γ_i allows a wide array of curvatures ranging from strong inverse S-shape to strong S-shape. The range chosen for δ_i implies that $w(0.5)$ is between $\frac{1}{11}$ and $\frac{3}{5}$, which is considered as a reasonable range given the previous findings in the literature and our nonparametric results suggesting strong underweighting at 0.5.

To facilitate hierarchical modelling, following Rouder and Lu (2005), Nilsson *et al.* (2011), and Scheibehenne and Pachur (2015), we used probit transformations of the individual level parameters γ_i and δ_i with linear linkages, i.e. $\gamma_i = 1.9 * \Theta(\gamma_i') + 0.1$ and $\delta_i = 1.4 * \Theta(\delta_i') + 0.1$ where Θ is the cumulative distribution function of the standard normal distribution. The probitized parameters γ_i' and δ_i' are assumed to come from normal distributions with $N(\mu_{\gamma}, \sigma_{\gamma})$ and $N(\mu_{\delta}, \sigma_{\delta})$ respectively. The priors of the group level means, μ_{γ} and μ_{δ}, were assumed to follow standard normal distributions, which result in uniform distributions with the aforementioned ranges when they are transformed back to rate scale. The priors of the group level standard deviations, σ_{γ} and σ_{δ}, were uniformly distributed ranging from 0 to 10.

The individual level noise parameter φ_i were assumed to come from a lognormal distribution. Similarly, to facilitate the hierarchical modeling, we used the logarithmic transformation of φ_i, i.e., $\varphi_i = exp(\varphi_i')$, where the prior of φ_i' assumed to follow $N(\mu_{\varphi}, \sigma_{\varphi})$. The group level mean, μ_{φ}, was assumed to be uniformly distributed ranging from -2.3 to 2.3, which results in a uniform distribution ranging from 0.1 to 10 in the exponential scale. The group level standard deviation σ_{φ} was uniformly

distributed ranging from 0 to 1.33. The upper bound of 1.33 was determined as the standard deviation of the prior distribution of the group level mean, $U(-2.3, 2.3)$, following Nilsson *et al.* (2011, pg. 88).

The MCMC algorithm was implemented in WinBUGS run through R software. Three chains, each with 60000 iterations were run, after a burn-in of 10000 iterations. To reduce the autocorrelation, only every 10th sample was recorded. Convergence was checked by Gelman-Rubin statistics, and by visual inspection of trace plots.

Figure A5.1 shows the posterior histograms for the group level mean parameters. Figure A5.2 shows the predictive performance of the estimations by comparing the median numbers of overweighting predicted by the posterior distributions of group-level parameters with the actual numbers of overweighting observed in our data. The model predictions match with the observed data for 0.2 and 0.9 in the DFD treatment, and for 0.05 and 0.1 in the DFE treatment. The predictions for the other probabilities were close to the actual data in the DFE treatment. The predictions for 0.05 and 0.8 in the DFD treatment indicated some misalignment with the actual data, although they performed well in the rest of the probabilities.

Figure A5.1 Posterior histograms for group level means

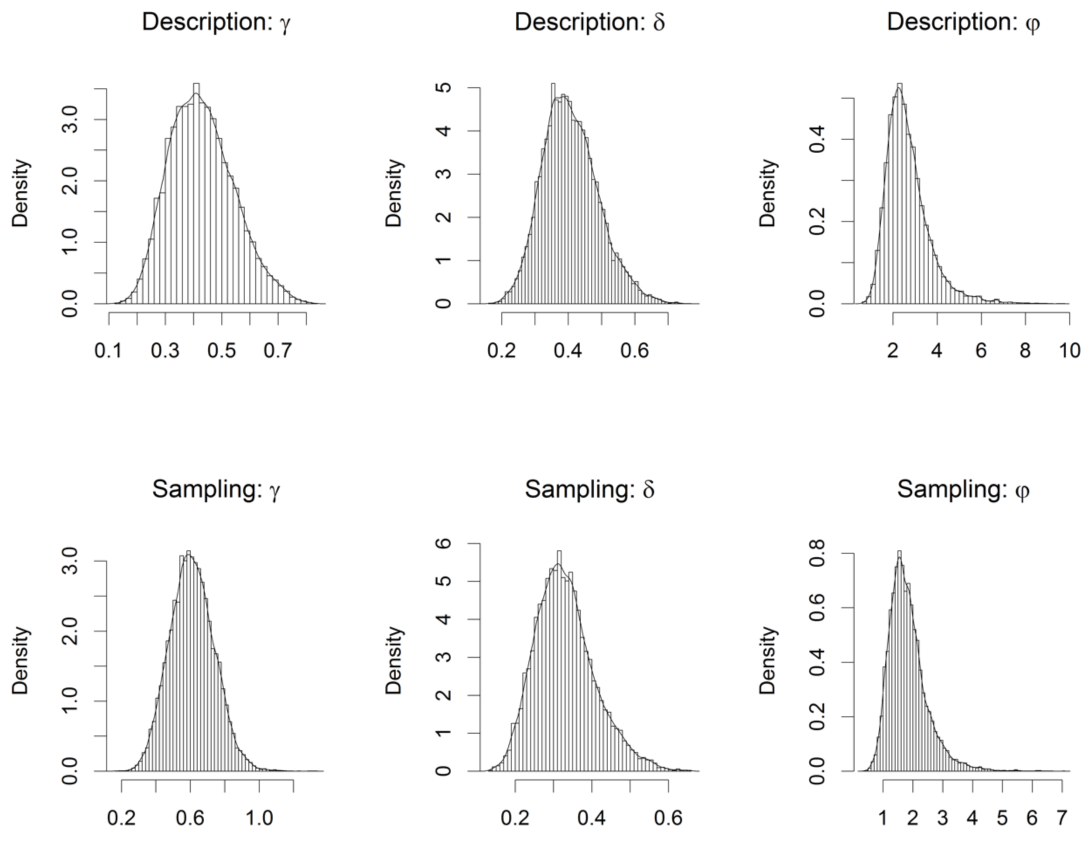

Figure A5.2 Posterior predictions based on group level parameters

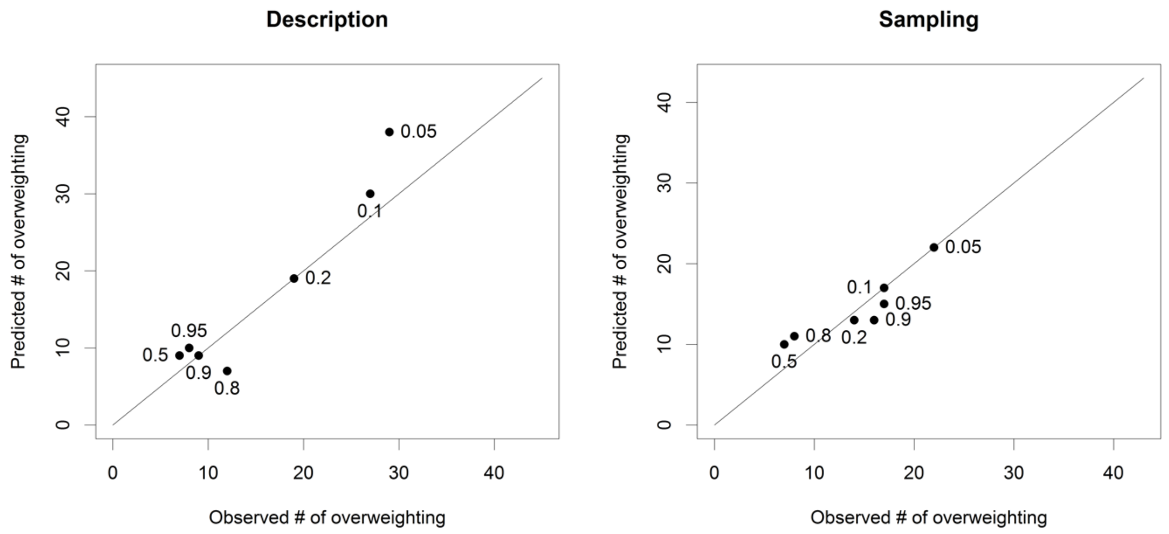

Online Appendix 6: Parametric Estimations with

Prelec's (1998) Compound Invariance Family

Prelec (1998)'s compound invariance family is given by $w(q) = e^{-\delta(-lnp)^{\gamma}}$. The parameter γ determines the curvature and captures the sensitivity towards changes in probabilities. Here, $\gamma < 1$ indicates inverse S-shape and likelihood insensitivity, and $\gamma > 1$ indicates S-shape and likelihood oversensitivity. The parameter δ determines the elevation, and it is an index of pessimism. Higher values of δ indicate less elevation and more pessimism. Table A6.1 and Figure A6.1 show the results of our Bayesian hierarchical estimations with this family.

Table A6.1. Group level mean parameters

	γ	δ
Description	0.382<br>[0.219, 0.593]	1.519<br>[1.292, 1.766]
Sampling	0.488<br>[0.298, 0.724]	1.670<br>[1.392, 1.912]
Gap	-0.106<br>[-0.393, 0.179]	-0.151<br>[-0.492, 0.213]

Notes: Estimated parameters are the means of the posterior distributions of the group level means. 95% credibility intervals are given in square brackets.

Figure A6.1. Probability weighting functions

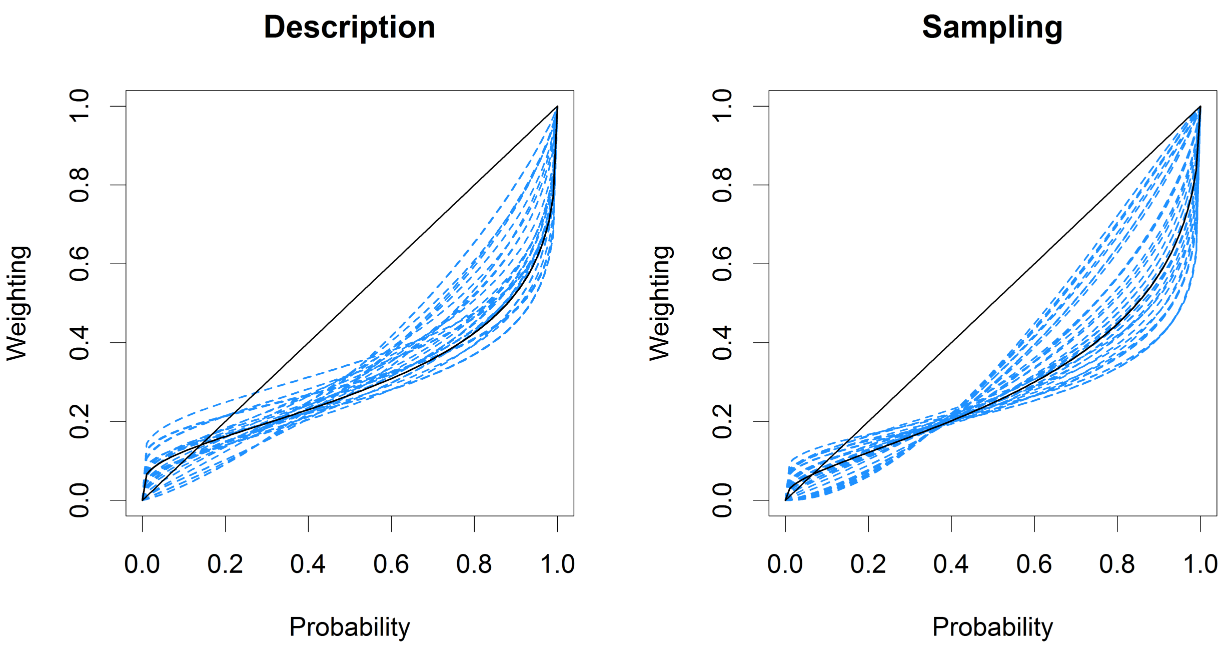

Estimations with Prelec's (1998) compound invariance family

The estimation procedure was the same. Prelec's compound invariance family is given by $w(q) = e^{-\delta(-lnp)^{\gamma}}$. The ranges of the prior distributions were from 0.1 to 2 for both γ_i and δ_i.

Figure A6.2 Posterior histograms for group level means

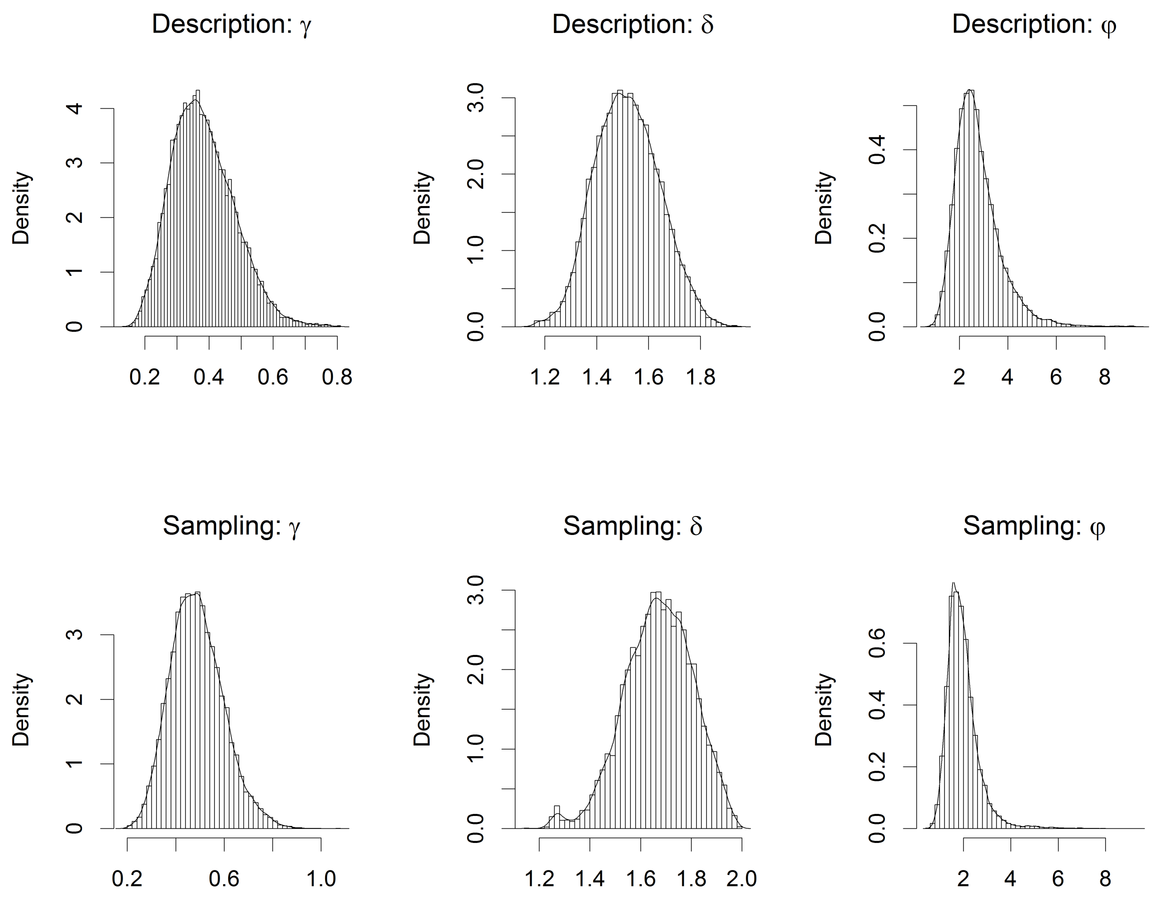

Figure A6.3 Posterior predictions based on group level parameters

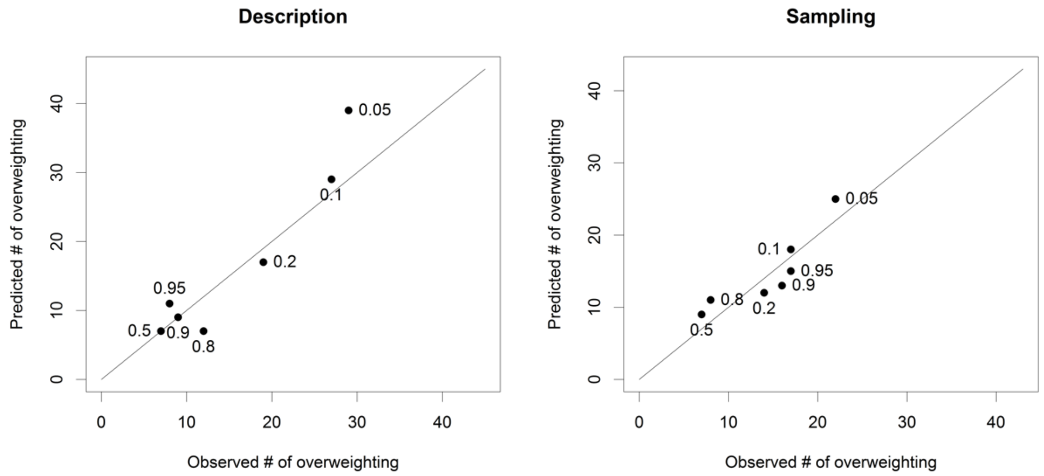

Online Appendix 7: Rounding problem

To check the potential problem caused by rounding, we address it in two ways:

1. Check if rounding in the calculation of s_q sequences can predict the choices in the second stage. The direction of rounding up or down cannot predict the choice in the second stage (Spearman correlation tests, $p>0.1$ for all).
2. Check if the direction of rounding is biased in the two groups of DFE and DFD.
   We compute the bias caused by rounding in two steps: 1. Calculate the numbers in lotteries without rounding. 2. Take the difference between the actual numbers before rounding (obtained in step 1) and after rounding (the numbers we used in the experiment). For example, if 92.2 was rounded to 92, then the difference would be 0.2.

**Prob.**	**DFD**		**DFE**		
	Median	Compare with 0 (p-values)	Median	Compare with 0 (p-values)	**Compare with each other (p-values)**
**0.05**	0.08	0.78	-0.03	1	0.53
**0.95**	0.05	0.83	0.01	1	0.45
**0.10**	0.12	0.83	-0.09	0.91	0.49
**0.90**	0.06	0.78	0.11	0.95	0.70
**0.20**	-0.02	0.87	-0.002	1	0.42
**0.80**	0.02	1	0.02	1	0.69
**0.50**	0.04	0.91	-0.04	0.84	0.53

Reported are the differences caused by rounding. We conducted Wilcoxon unpaired tests.

Online Appendix 8: The notes

As the sampled outcomes were both subject- and question-specific by our two-stage experimental design, we were able to identify the choice questions on the notes. We observed that in total 28 subjects took notes in at least two out of seven choice questions. Among these, for 19 subjects, we observed notes for all the sampled outcomes in every choice question, and for 13 subjects, we observed some counting of frequencies in every choice question. Here, we present some examples of the notes taken by our subjects. The full notes can be found here:

https://www.dropbox.com/s/yxttzvr6tdl8jr1/notes.pdf?dl=0

The notes that were taken by Subject 3107 in Example 1 suggest a very comprehensive counting of the outcome frequencies. The notes taken by Subject 3404 in Example 2 also suggest a process of counting the frequencies for every choice question.

Example 1. The notes of Subject 3107

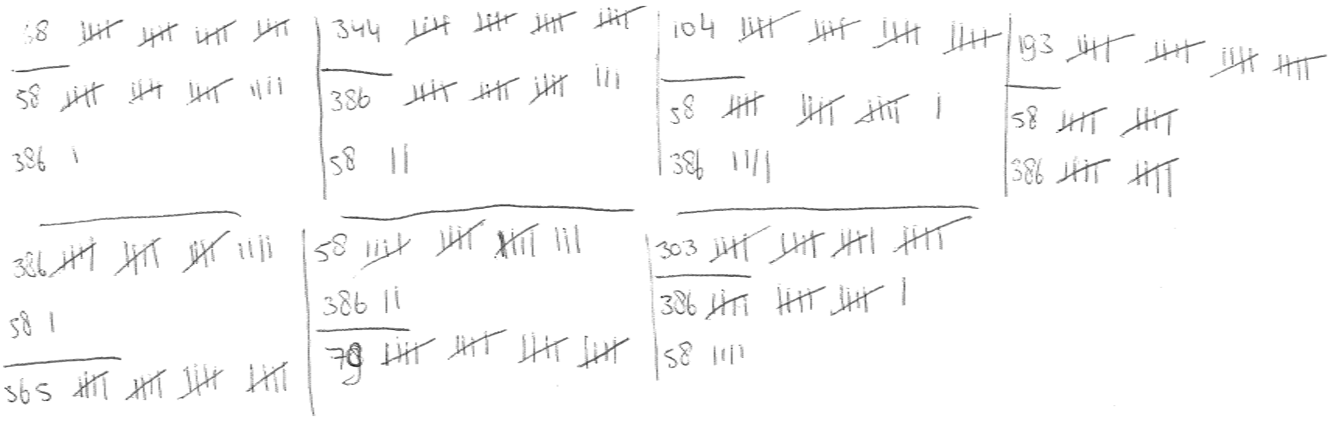

Example 2. The notes of Subject 3404

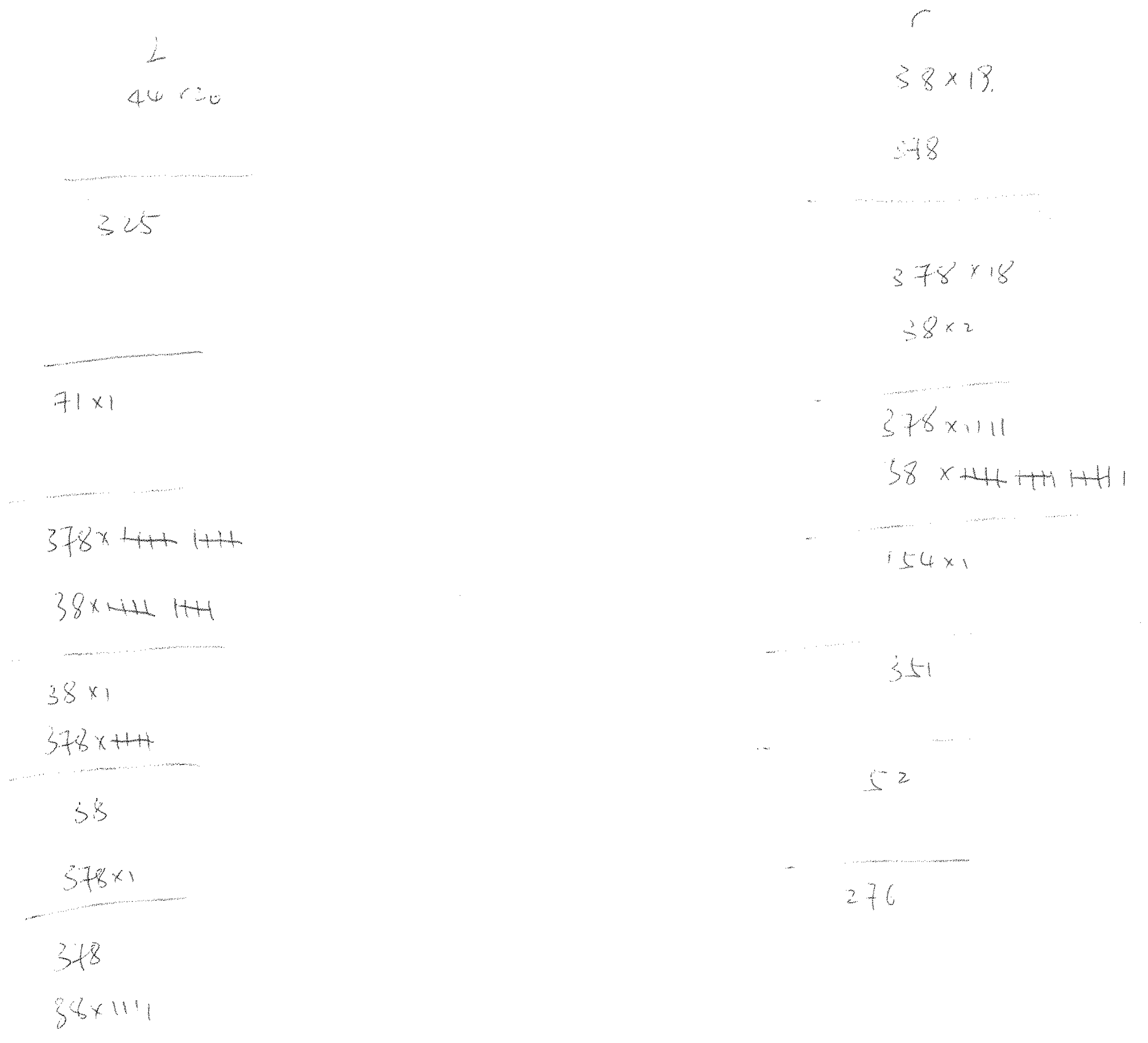

Example 3 presents the notes taken by Subject 3409. Although we detect some notes taken by this subject for every choice question, we observe that some of his or her notes mention only about the sampled outcomes but not about their frequencies. For example, the notes concerning the choice question with 95% probability (indicated inside the square) show only the sampled outcomes but not the observed frequencies of those.

Example 3. The notes of Subject 3409

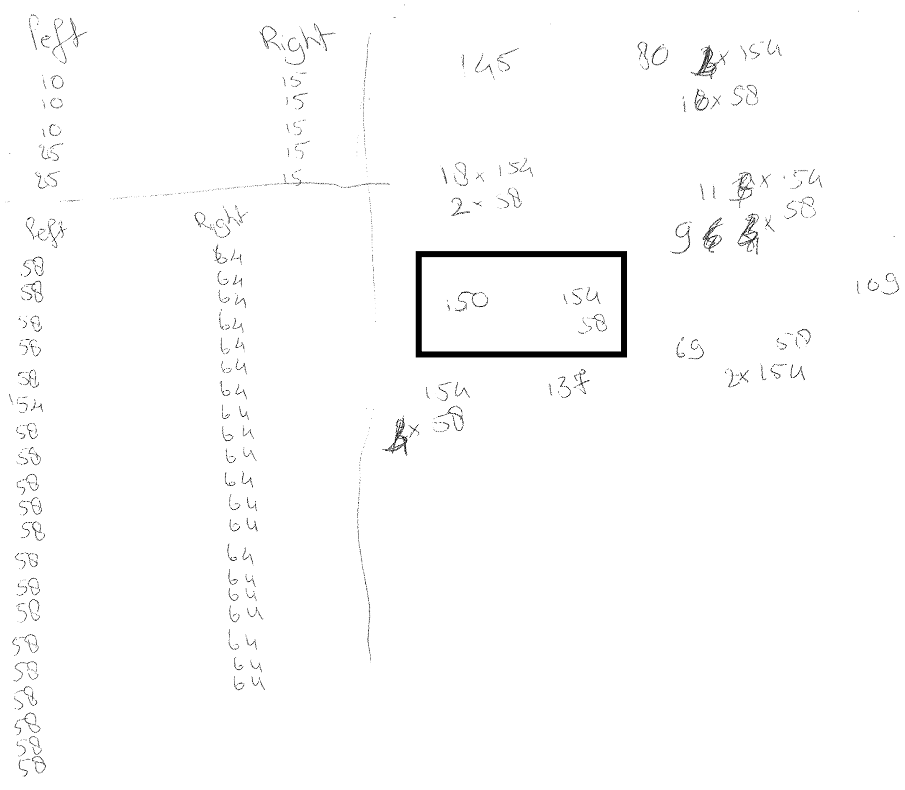

Among all the notes, we detected in total of six cases where the notes suggested some potential mistakes in counting the outcome frequencies. For example, in the notes taken by Subject 3311 presented in Example 4, the frequency of the rare outcome 50 was indicated as six, whereas the actual frequency of this outcome was four.

Example 4. One note of Subject 3311

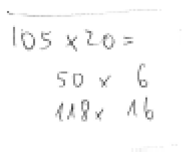

An exploratory analysis of the impact of note-taking during the experiment did not detect a significant effect on choice behavior. In a repeated-measures logistic regression that pooled the choices concerning the weighting of six small probabilities, the odds ratio of overweighting small probabilities between those who took notes and those who did not was estimated as 1.186. This indicated slightly higher odds of overweighting small probabilities among subjects who took notes than among those who did not. However, this ratio did not differ from one (p-value=0.618). Here, the independent variable of note-taking was identified with 28 subjects who took some notes in any of the choice questions. When we

identify note-taking with 19 subjects who took notes in every choice question, the odds ratio is estimated as 1.244. Nevertheless, this ratio did not differ from one either (p-value=0.539).

Online Appendix 9: Recency effects

We examine the role of recency in our data as follows. We define recency in situations where the majority of the rare outcome observations (i.e., 1 out of 1 for 0.05, 2 out of 2 for 0.10, and 3 out of 4 for 0.20) were observed at the last half of the sequence. We estimate the impact of recency by running random effects logistic regressions, where the dependent variable is over- or under-weighting of probabilities and the independent variable is a dummy variable for recency. The good- and bad-event probabilities are pooled in the estimations summing up 258 observations (6 choices involving rare outcomes for 43 subjects). The odds ratio is estimated as 1.133 indicating a slightly higher odds of overweighing when the rare outcome is observed recently. However, this ratio did not differ from one (p-value=0.673). Therefore, it did suggest a significant recency effect.

References

Abdellaoui, M., O. L'Haridon and C. Paraschiv (2011). 'Experienced vs. Described Uncertainty: Do We Need Two Prospect Theory Specifications?', Management Science, vol. **57**, pp. 1879-1895.

Erev, I., E. Ert, A. E. Roth, E. Haruvy, S. M. Herzog, R. Hau, R. Hertwig, T. Stewart, R. West and C. Lebiere (2010). 'A choice prediction competition: Choices from experience and from description', Journal of Behavioral Decision Making, vol. **23**, pp. 15-47.

Frey, R., R. Mata and R. Hertwig (2015). 'The role of cognitive abilities in decisions from experience: Age differences emerge as a function of choice set size', Cognition, vol. **142**, pp. 60-80.

Glöckner, A., B. E. Hilbig, F. Henninger and S. Fiedler (2016). 'The reversed description-experience gap: Disentangling sources of presentation format effects in risky choice.', Journal of Experimental Psychology: General, vol. **145**, pp. 486.

Goldstein, W. M. and H. J. Einhorn (1987). 'Expression theory and the preference reversal phenomena.', Psychological review, vol. **94**, pp. 236.

Hau, R., T. J. Pleskac, J. Kiefer and R. Hertwig (2008). 'The description–experience gap in risky choice: the role of sample size and experienced probabilities', Journal of Behavioral Decision Making, vol. **21**, pp. 493-518.

Hertwig, R., G. Barron, E. U. Weber and I. Erev (2004). 'Decisions from Experience and the Effect of Rare Events in Risky Choice', Psychological Science, vol. **15**, pp. 534-539.

Kellen, D., T. Pachur and R. Hertwig (2016). 'How (in)variant are subjective representations of described and experienced risk and rewards?', Cognition, vol. **157**, pp. 126-138.

Kemel, E. and M. Travers (2016). 'Comparing attitudes toward time and toward money in experience-based decisions', Theory and Decision, vol. **80**, pp. 71-100.

Luce, R. D. (1959). Individual Choice Behavior a Theoretical Analysis: John Wiley and sons.

Nilsson, H., J. Rieskamp and E.-J. Wagenmakers (2011). 'Hierarchical Bayesian parameter estimation for cumulative prospect theory', Journal of Mathematical Psychology, vol. **55**, pp. 84-93.

Prelec, D. (1998). 'The probability weighting function', Econometrica, pp. 497-527.

Regenwetter, M. and M. M. Robinson (2017). 'The construct–behavior gap in behavioral decision research: A challenge beyond replicability.', Psychological review, vol. **124**, pp. 533.

Rouder, J. N. and J. Lu (2005). 'An introduction to Bayesian hierarchical models with an application in the theory of signal detection', Psychonomic Bulletin & Review, vol. **12**(**4**), pp. 573-604.

Scheibehenne, B. and T. Pachur (2015). 'Using Bayesian hierarchical parameter estimation to assess the generalizability of cognitive models of choice', Psychonomic Bulletin & Review, vol. **22**, pp. 391-407.

Ungemach, C., N. Chater and N. Stewart (2009). 'Are Probabilities Overweighted or Underweighted When Rare Outcomes Are Experienced (Rarely)?', Psychological Science, vol. **20**, pp. 473-479.
